# RBM17 promotes hepatocellular carcinoma progression by regulating lipid metabolism and immune microenvironment: implications for therapeutic targeting

**DOI:** 10.1038/s41420-025-02642-2

**Published:** 2025-07-23

**Authors:** Zengbin Wang, Jiayu Liu, Yiting Lai, Qing Zhong, Qian Su, Linqing Wu, Zhihong Wang, Zhuting Fang

**Affiliations:** 1 Department of Immunology, School of Basic Medical Sciences, Fujian Medical University, Fuzhou, 350122 China; 2 Department of Physiology and Pathophysiology, School of Basic Medical Sciences, Fujian Medical University, Fuzhou, 350122 China; 3 College of Integrative Medicine, Fujian University of Traditional Chinese Medicine, Fuzhou, 350100 China; 4 Department of Gastric Surgery, Fujian Medical University Union Hospital, Fuzhou, 350001 China; 5 Shengli Clinical Medical College of Fujian Medical University, Fuzhou, 350122 China; 6 Department of Hematology, Fujian Provincial Hospital, Fuzhou, 350001 China; 7 Fuzhou University Affiliated Provincial Hospital, Fuzhou, 350001 China; 8 Department of Oncology and Vascular Interventional Therapy, Clinical Oncology School of Fujian Medical University, Fujian Cancer Hospital (Fujian Branch of Fudan University Shanghai Cancer Center), Fuzhou, 350014 China; 9 Department of Interventional Radiology, Fujian Provincial Hospital, Shengli Clinical Medical College of Fujian Medical University, Fuzhou, 350001 China

**Keywords:** Cancer metabolism, Immune evasion

## Abstract

Variable splicing (AS) plays important roles in tumor progression. However, the role of the AS factor RBM17 in the progression of hepatocellular carcinoma (HCC) has not yet been elucidated. We used label-free proteomics, single-cell sequencing (scRNA-seq), high throughput sequencing, flow cytometry (FCM), liquid Chromatography-tandem mass spectrometry (LC‒MS/MS), multiparametric immunofluorescence (mIF) and chromatin immunoprecipitation (Chip), to explore the relationship between RBM17 regulation of HCC cell lipid metabolism and the immune microenvironment. Our findings revealed that RBM17 is significantly overexpressed in HCC tissue and is positively correlated with poor prognosis. We found a positive correlation between RBM17 expression and M2 macrophage infiltration. Mechanistically, RBM17 promotes M2 macrophage infiltration by inducing taurocholic acid (T-CA) production, which is achieved through enhancing exon exclusion of CSAD precursor mRNA. Additionally, RBM17 modulates fatty acid metabolism and CD8^+^ T cell infiltration by regulating exon skipping in HACD3 precursor mRNA. Furthermore, RUNX1 activates RBM17 expression and regulates downstream CSAD/T-CA and HACD3/FFA signaling. Importantly, targeting RBM17 can prevent HCC progression, suggesting its potential as a therapeutic target for HCC. Our findings provide new insights into the mechanisms underlying immune cell infiltration and metabolism in HCC and identify RBM17 as a promising therapeutic target.

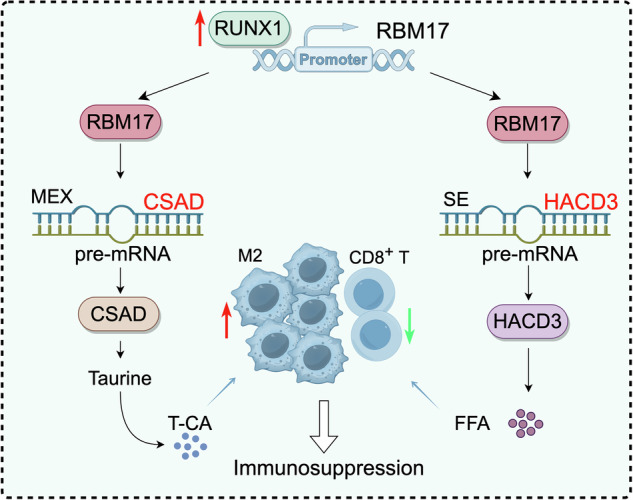

## Introduction

Hepatocellular carcinoma (HCC) is characterized by a high incidence rate, high mortality rate, difficulty in early diagnosis, and insensitivity to chemotherapy. Due to complex molecular mechanisms such as genomic complexity and epigenetic diversity, HCC exhibits high heterogeneity at both the clinical and molecular levels [1]. Currently, the treatment options for HCC primarily include tyrosine kinase inhibitors, such as sorafenib and lenvatinib. Immunotherapy is a new area in the treatment of HCC; however, when patients have weakened immune function, it may not be possible to improve their quality of life or prognosis through immunotherapy. Additionally, there are numerous obstacles to the antitumor immune response in HCC cells, including abnormalities in the cellular and humoral immune systems, changes in T-cell surface markers, and other factors. These factors contribute to the complexity and diversity of the pathophysiological process of HCC, making immunotherapy a highly challenging approach [2–4]. This therapeutic impasse underscores the urgent need for novel therapeutic targets that can simultaneously disrupt metabolic addiction and reverse immune exhaustion.

Alternative splicing (AS), a crucial posttranscriptional regulatory mechanism [5], plays a pivotal role in the development and progression of HCC [6, 7]. AS factors can modulate the glycolytic and lipid metabolism of tumor cells [8–10]. For instance, the AS factor SF3B3 collaborates with the RNA-binding protein RALY to inhibit MTA1-S expression, thereby inducing the expression of cholesterol synthesis genes and promoting HCC cell proliferation [10]. Inhibitors of the AS factor Rbm39 have been shown to enhance the efficacy of immune checkpoint inhibitors [11]. A notable example is aryl sulfonamides like indisulam, which target RBM39 for ubiquitination-mediated degradation, exhibiting anticancer effects in HCC [12]. The AS factor Rbm17 plays a critical role in the progression of colorectal cancer and gliomas [13, 14]. In addition, Rbm17 is a predictive biomarker for the efficacy of immune checkpoint inhibitors (ICIs) in the treatment of NSCLC patients with low PD-L1 expression [15]. However, its dual regulatory roles in HCC metabolic reprogramming and immune modulation remain unexplored.

Here, we have identified two previously unrecognized lipid regulatory axes mediated by RBM17, namely CSAD/T-CA and HACD3/FFA, which ultimately lead to immune suppression in HCC. We aimed to elucidate the molecular mechanisms underlying the regulation of HCC cell lipid metabolism and immune suppression by RBM17 by utilizing techniques such as label-free proteomics, scRNA-seq, RNA-seq, LC‒MS/MS, Chip, FCM and mIF. This research identified RBM17 as a therapeutic target for HCC, providing theoretical and experimental evidence for the promotion of RNA splicing as a novel therapeutic approach.

## Results

### The expression of AS factor is significantly enhanced in HCC tissue

**Fig. 1:**
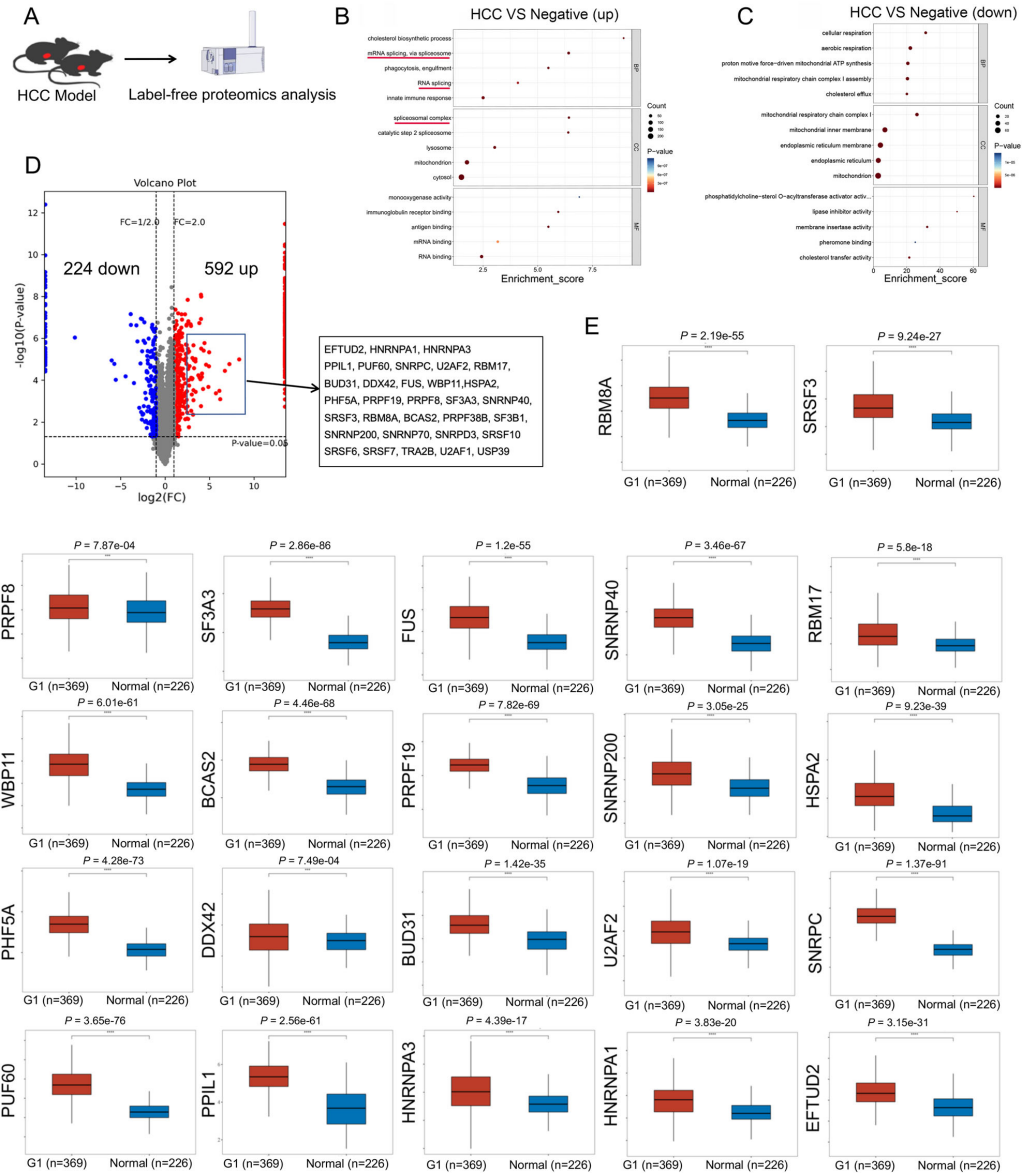
Increased expression of AS factors in HCC tissue. **A** The schematic diagram represents the constructed DEN induced HCC model for label free proteomics analysis. Negative represents the control group, also known as the “PBS” group. HCC represents the model group. *n* = 3. **B**, **C** Bubble plots for the top 15 enriched genes and the top 15 downregulated genes. **D** Volcano map. The horizontal axis of the volcano map is log2 (FC), and the further away the value is from point 0, the greater the difference. The right side shows an increase, and the left side shows a decrease. The vertical axis is -log10 (*P* value), and the farther the vertical axis value is from point 0, the greater the difference. The screening method for 32 upregulated AS factors is: Foldchange ≥ 2 and *P*-value < 0.05. **E** This figure illustrates the boxplot depicting the expression distribution of target genes across tumor tissue and normal tissue. The statistical significance between the two sets of samples was assessed using the *Wilcoxon* test.

To elucidate the expression of AS factors in HCC tissue, we developed a DEN induced HCC model and employed label-free proteomics analysis (Fig. 1A). GO enrichment analysis of the differentially expressed proteins revealed an increase in AS splicing signals (Fig. 1B, C). Compared with the control group, the HCC group displayed upregulated expression of 592 proteins and downregulated expression of 224 proteins (Fig. 1D). Notably, the expression levels of 32 AS factor proteins were markedly increased (Fig. 1D). Furthermore, we analyzed the TCGA-LIHC dataset and found that 22 of the 32 AS factors mentioned above (EFTUD2, HNRNPA1, HNRNPA3, PPIL1, PUF60, SNRPC, U2AF2, RBM17, BUD31, DDX42, FUS, HSPA2, PHF5A, PRPF19, PRPF8, SF3A3, SNRNP200, SNRNP40, SRSF3, WBP11, RBM8A and BCAS2) were highly expressed in HCC tissue compared to normal liver tissue (Fig. 1E). These results collectively indicate a significant upregulation of AS factor expression in HCC tissue.

### RBM17 is associated with poor prognosis in HCC patients

**Fig. 2:**
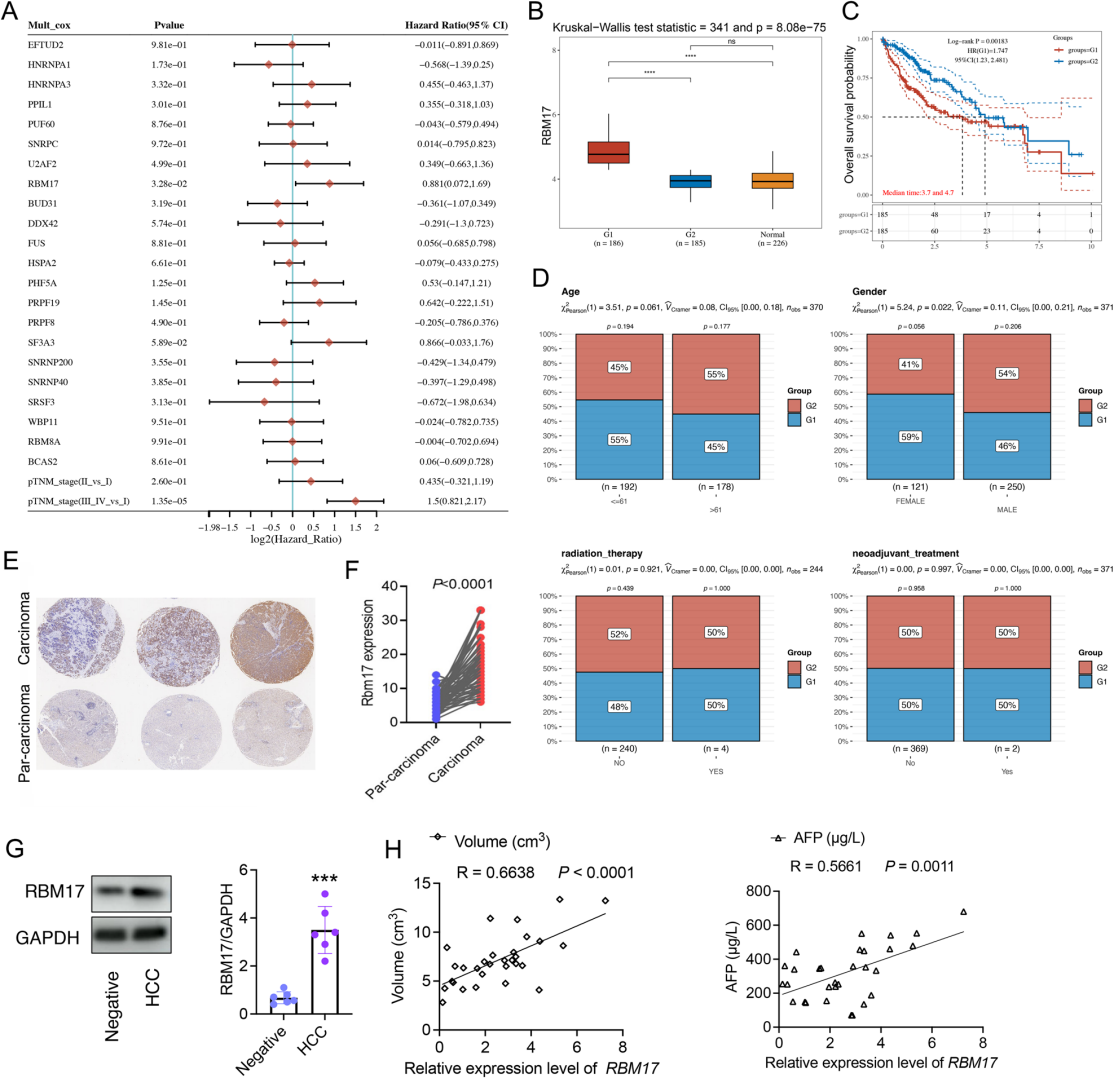
The AS factor RBM17 is associated with poor prognosis in HCC patients. **A** Multivariate Cox analysis of gene expression and clinical characteristics of 22 AS factors, including *p*-values, risk coefficients (HR), and confidence intervals. **B**, **C** Comparing the relationship between high and low expression of RBM17 and prognosis of HCC patients. G1 represents high expression of RBM17, and G2 represents low expression of RBM17. **D** The distribution of clinical features in high and low expression of RBM17. The horizontal axis represents different clinical information, and the vertical axis represents the percentage of clinical information contained in the corresponding grouped samples. We analyzed the significance *p*-values between different groups using chi square test. **E** IHC analysis of RBM17 expression in HCC tissue microarrays for carcinoma and par-carcinoma. **F** Comparing the expression of RBM17 between carcinoma and par-carcinoma. A paired *t-*test was used for data analysis. *n* = 75. **G** Immunoblotting was used to detect the expression levels of RBM17 protein in primary liver tissue parenchymal cells of the negative and HCC groups. The data were analyzed using *t-*tests. *n* = 6. **H** Pearson correlation analysis of the correlation between RBM17 and tumor volume and AFP values. *n* = 30. ns represents no significant difference. *P < 0.05, **P < 0.01, ***P < 0.001, ****P < 0.0001.

To assess the connection between AS factors and HCC prognosis, we conducted a multivariate Cox proportional hazards regression analysis and used the “Forestplot” package to create a forest map. The results showed that RBM17 was the most significant independent risk factor (Fig. 2A). To analyze the relationship between the differential expression levels of RBM17 and the prognosis of HCC patients, RBM17 was divided into two groups: high and low expression (Fig. 2B). HCC patients with high RBM17 expression had a poor prognosis (Fig. 2C). In addition, we further compared the clinical feature distribution of high and low expression of RBM17, and found that the gender characteristics of the RBM17 high expression group showed significant differences compared to the RBM17 low expression group, while there were no significant differences in age, radiotherapy, and neoadjuvant therapy (Fig. 2D). Based on these findings, we employed immunohistochemistry (IHC) to assess RBM17 expression in HCC tissue microarrays (Fig. 2E). The expression level of RBM17 was greatly elevated in carcinoma tissue compared to that in para-carcinoma tissue (Fig. 2E, F). We extracted primary parenchymal cells from liver tissue of DEN induced HCC mice and found that the expression level of RBM17 protein was significantly upregulated in the HCC group (Fig. 2G). We also analyzed the liver tissue of HCC patients and found that the expression of RBM17 was positively correlated with tumor size and AFP value (Fig. 2H), indicating its potential role in HCC progression and poor prognosis.

### The expression level of RBM17 is positively correlated with M2 macrophage infiltration

**Fig. 3:**
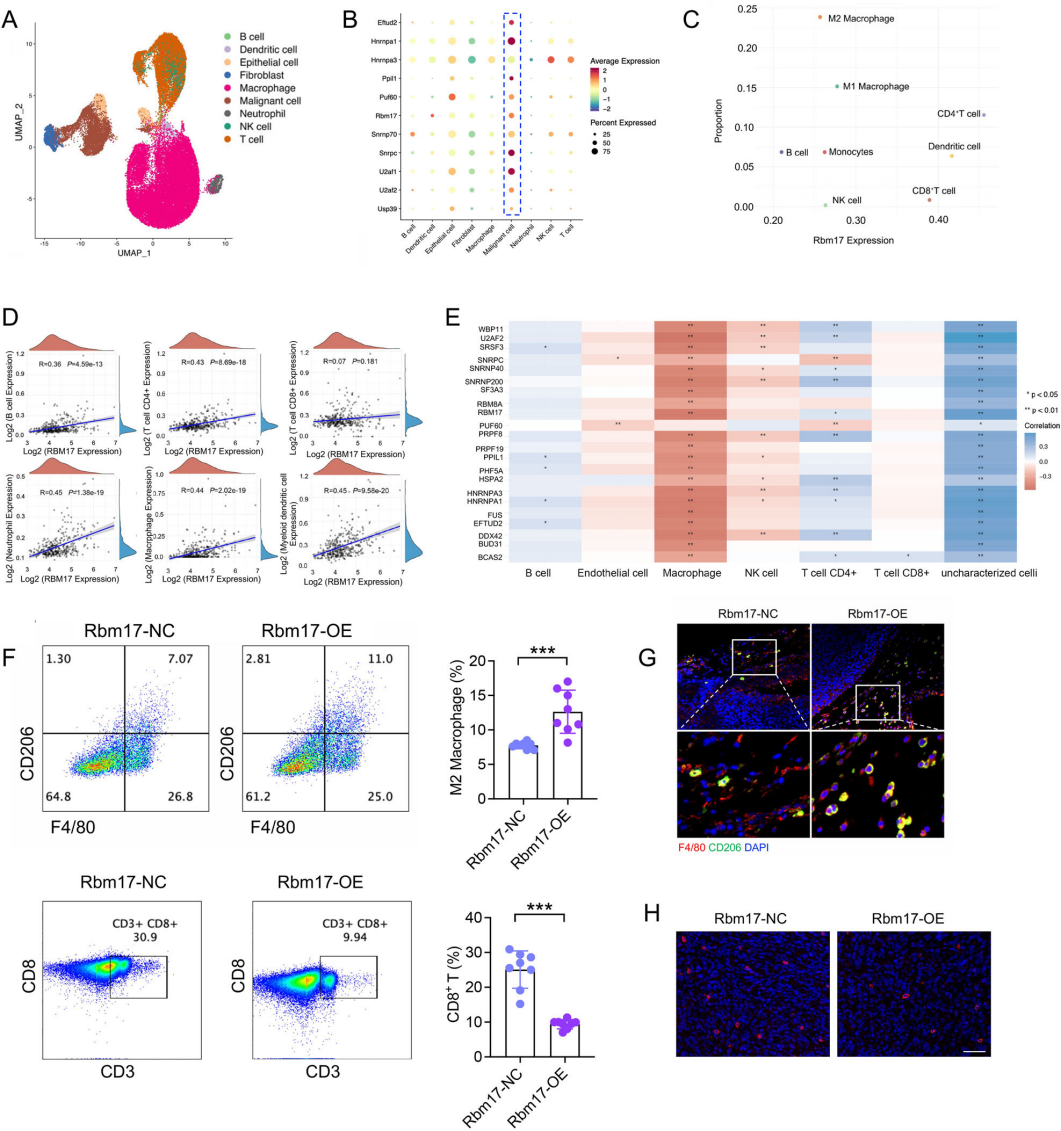
The expression of RBM17 is positively correlated with the infiltration level of M2 macrophages. **A** We used the unsupervised dimensionality reduction algorithm UMAP to create a two-dimensional graph, dividing immune cells into six groups: B cells, dendritic cells, macrophages, neutrophils, natural killer cells and T cells. **B** The expression of RBM17 in various cell populations. **C** The correlation between RBM17 expression and the proportion of immune cells. **D** We downloaded RNA-seq data and corresponding clinical information for LIHC patients from the TCGA database. The data were analyzed through the R software package ggstatsplot. Spearman correlation analysis was used to assess the correlation between nonnormally distributed quantitative variables. **E** The relationship between the expression of 22 AS factors and macrophage infiltration. **F** FCM analysis of the effect of overexpression of RBM17 on the infiltration of M2 macrophages and CD8^+^ T cells in the microenvironment. The data were analyzed using unpaired *t-*tests. *n* = 8. **G**, **H** The infiltration level of M2 macrophages (F4/80^+^ CD206^+^) and CD8^+^ T cells in the subcutaneous HCC model was determined using mIF. *P < 0.05, **P < 0.01, ***P < 0.001.

To assess the expression and functional role of RBM17 in HCC, we constructed a subcutaneous HCC model. We extracted cancer tissue cells for scRNA-seq and performed dimensionality reduction clustering analysis on the obtained data. We also used the unsupervised dimensionality reduction algorithm (UMAP) to construct a two-dimensional graph and divided immune cells into 6 groups: B cells, dendritic cells, macrophages, neutrophils, natural killer cells and T cells (Fig. 3A). Further analysis revealed that RBM17 was mainly expressed in malignant cells, while it was expressed at low levels in immune cells (Fig. 3B). Therefore, we conducted an in-depth analysis of the relationship between Rbm17 expression and immune cell infiltration. The scRNA-seq data showed a positive correlation between Rbm17 expression and the proportions of M2 macrophages and CD4^+^ T cells (Fig. 3C). In addition, we analyzed the TCGA-LIHC data and discovered that the expression of RBM17 was positively correlated with the infiltration of macrophages (Fig. 3D). More importantly, these AS factors, including RBM17, are positively correlated with macrophages (Fig. 3E).

To analyze whether the growth effect of RBM17 on HCC is related to immunity, we constructed a Hep53.4 cell line overexpressing RBM17. We found that overexpression of RBM17 did not promote HCC growth in immunocompromised Rag1-KO mice (Fig. S1A, S1B). Furthermore, we found that overexpression of Rbm17 significantly promoted HCC growth in immunocompetent C57BL/6 N mice, indicating that RBM17 may promote HCC progression by affecting immune cells (Fig. S1C). To test the effect of RBM17 on immune microenvironment cells, we established a mouse subcutaneous HCC model. FCM analysis showed that overexpression of Rbm17 promotes the infiltration of M2 macrophages and inhibits the infiltration of CD8^+^ T cells (Fig. 3F, Fig. S2). The mIF experiment also demonstrated this effect (Fig. 3G, H). These findings illuminate that the overexpression of RBM17 in HCC cells facilitates the infiltration of M2 macrophages cells, while simultaneously inhibiting the infiltration of CD8^+^ T cells.

### RBM17 promotes M2 macrophage infiltration by inducing T-CA production

**Fig. 4:**
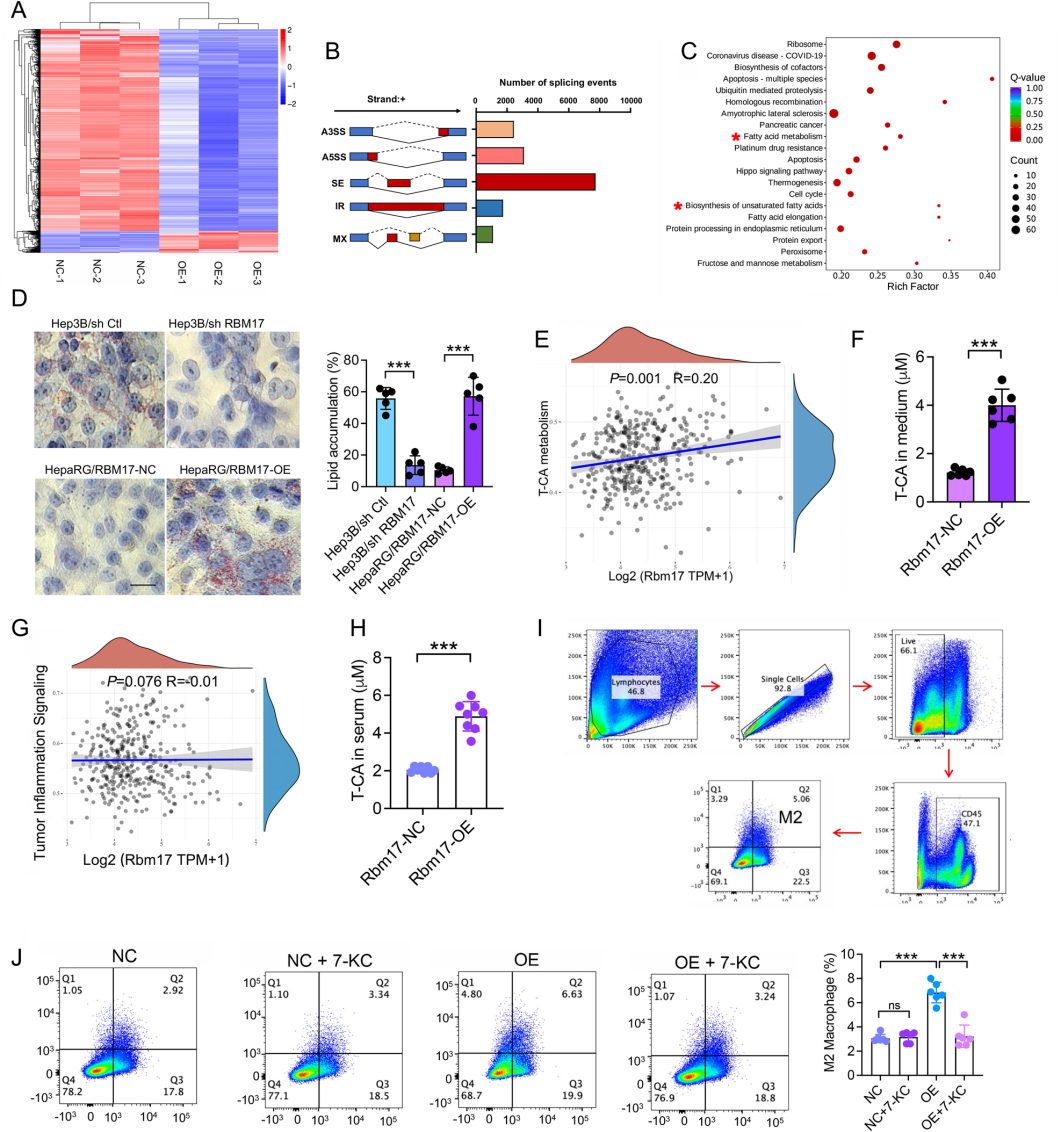
RBM17 promotes M2 macrophage infiltration by inducing T-CA secretion. **A** Heatmap of gene expression in HepaRG/RBM17-NC and HepaRG /RBM17-OE. **B** 13397 splicing events regulated by RBM17 (judged based on diff absolute value greater than zero and [bayes_factor ≥ 1]). **C** KEGG enrichment analysis of splicing events affected by RBM17. **D** Detection of cellular lipid deposition by Oil Red O staining. The data were analyzed using unpaired *t-*tests. *n* = 3. **E** Biological function analysis of RBM17. The *X*-axis in the figure represents the expression of RBM17, the *Y*-axis represents the pathway score, the density curve on the right represents the distribution trend of the pathway score. **F** LC‒MS detection of the T-CA concentration in cell culture supernatant. The data were analyzed using unpaired *t-*tests. *n* = 6. **G** Spearman correlation analysis of the relationship between RBM17 expression and inflammatory signaling in LIHC data from TCGA. **H** LC‒MS detection of the serum T-CA concentration in mice with subcutaneous HCC. The data were analyzed using *t-*tests. *n* = 8. **I**, **J** The infiltration level of M2 macrophages in the subcutaneous HCC model was determined using FCM. The data were analyzed using unpaired *t-*tests. ns represents no significant difference. *n* = 6. *P < 0.05, **P < 0.01, ***P < 0.001.

We continued to evaluate how high RBM17 expression in HCC cells affects M2 macrophage infiltration. We analyzed the impact of RBM17-overexpression (RBM17-OE) on splicing events through high throughput sequencing. The heatmap showed the degree of gene expression differences between samples (Fig. 4A). A total of 13397 splicing events regulated by RBM17 were identified, including 7 types of splicing, with exon skipping (SE) accounting for the vast majority (Fig. 4B). These splicing events involved 4571 genes. Enrichment analysis revealed that splicing events were mainly involved in fatty acid metabolism, unsaturated fatty acid biosynthesis (Fig. 4C). Previous studies have shown that bile acids (BAs), an important component of lipid metabolism, can promote macrophage M2 polarization in HCC [16]. We speculated that RBM17 promotes the polarization of M2 macrophages by promoting BAs secretion. We next constructed RBM17-overexpression and RBM17-knockdown cell lines using HepaRG and Hep3B, respectively. Oil red O staining showed that RBM17 overexpression promoted lipid deposition, while RBM17 knockdown had the opposite effect (Fig. 4D). Spearman correlation analysis revealed a positive correlation between RBM17 expression and the metabolism of a primary conjugated BAs, taurocholic acid (T-CA) [16], which is involved in lipid metabolism (Fig. 4E). To clarify whether RBM17 affects the metabolism of T-CA, we performed non targeted metabolic sequencing on HepaRG/RBM17-NC and HepaRG/RBM17-OE cells. We screened differential metabolites according to the criteria of VIP > 1.0, FC > 1.5, and *P*-value < 0.05. As a result, it was found that seven metabolites were upregulated in the RBM17 overexpression group, and all seven metabolites belonged to bile acids, alcohols, and derivatives, including T-CA (Fig. S3A–S3C). LC‒MS analysis showed a significant increase in the T-CA concentration in the supernatant of cells overexpressing RBM17 (Fig. 4F), indicating that RBM17 promotes T-CA production from HCC cells.

HCC cell-derived T-CA can activate M2 macrophage polarization [16]. Bioinformatics analysis revealed no correlation between RBM17 expression and HCC-related inflammation (Fig. 4G), providing that RBM17 may not affect the secretion of inflammation-related cytokines. Further LC‒MS analysis of mouse subcutaneous HCC serum also showed that RBM17 overexpression increased T-CA secretion (Fig. 4H). In addition, we also confirmed that the infiltration of M2 macrophages induced by RBM17-OE can be inhibited by T-CA inhibitors (7-KC) in vivo experiment (Fig. 4I, J). To clarify whether the effect of T-CA on M2 polarization is related to FXR activation of macrophages, we conducted in vitro experiments. The qRT-PCR results indicated that T-CA treatment significantly induced mRNA transcription levels of *FXR* and *CD206* in THP1 cells (Fig. S4A, S4B). Hepatocyte-derived supernatant and PMA-induced THP1 cells were cocultured in vitro. The expression of CD206 in THP1 cells in the RBM17-OE cell group was significantly greater compared with that in the RBM17-NC cell group, but this effect was inhibited by 7-KC (Fig. S4C, S4D). Additionally, qRT-PCR results also suggestd that the supernatant of cells overexpressing RBM17 can activate FXR expression (Fig. S4E). Therefore, we speculated that RBM17 promotes the secretion of T-CA, thereby inducing the polarization of M2 macrophages in the microenvironment.

### RBM17 induces T-CA generation by enhancing exon exclusion of CSAD precursor mRNA

**Fig. 5:**
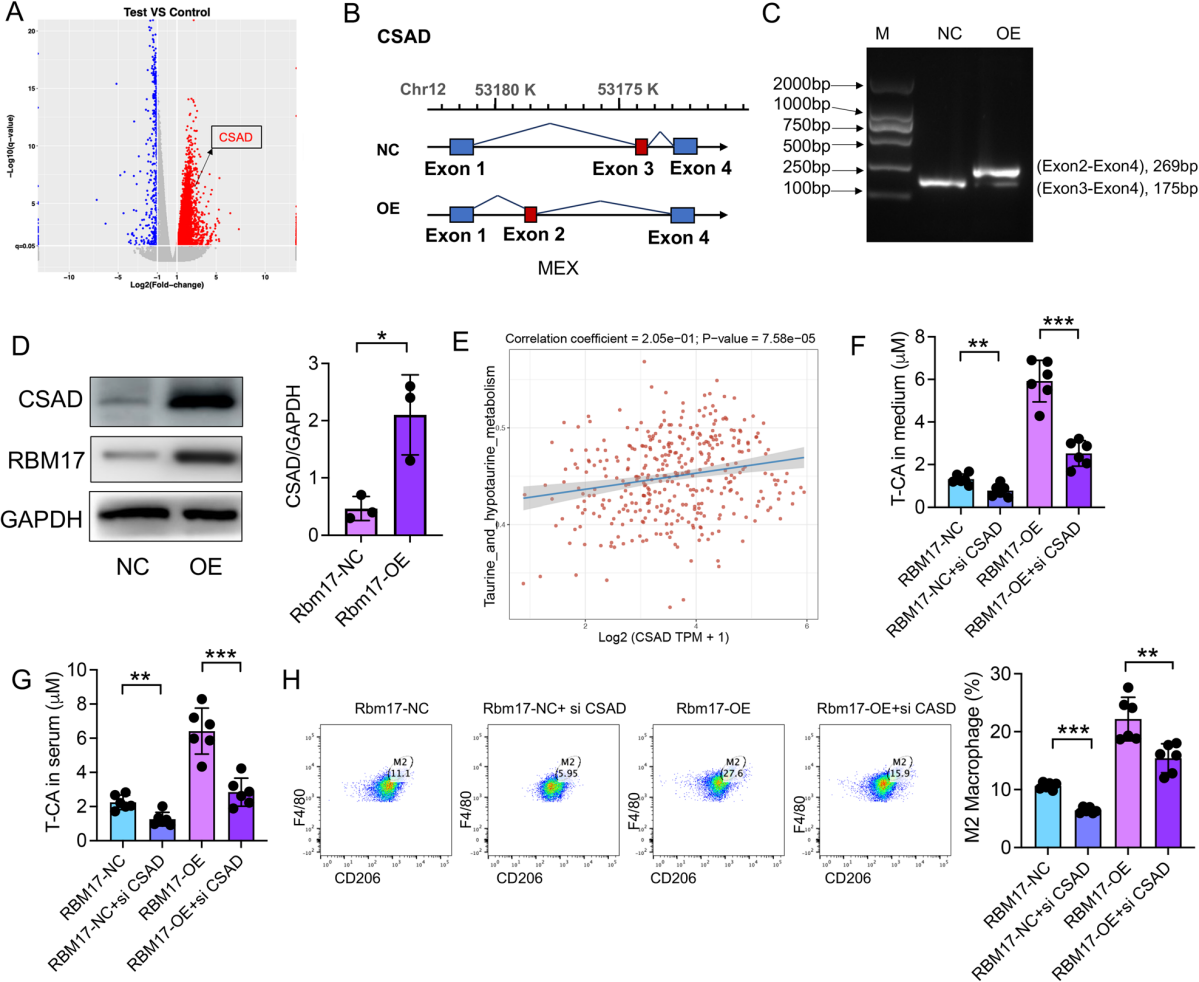
RBM17 promotes the expression of the CSAD gene and stimulates the production of T-CA by facilitating the exclusion of exons in the precursor mRNA of the CSAD gene. **A** Volcanic map of differentially expressed genes. **B** The CSAD gene undergoes exon exclusion after overexpression of RBM17. **C** PCR validation of CSAD AS events. Exon2-Exon4 represents primers designed from the second exon to the fourth exon, while Exon3-Exon4 represents primers designed from the third exon to the fourth exon. **D** Immunoblot detection of protein expression levels of CSAD and RBM17. Data was analyzed using unpaired *t-*tests. *n* = 3. **E** Spearman correlation analysis of the relationship between CSAD expression and taurine metabolism in LIHC data from TCGA. **F** LC‒MS detection of the T-CA concentration in cell culture supernatant. The data were analyzed using unpaired *t-*tests. *n* = 6. **G** LC‒MS detection of the serum T-CA concentration in mice with subcutaneous HCC. **H** The infiltration level of M2 macrophages in the subcutaneous HCC model was determined using FCM. The data were analyzed using *t-*tests. *n* = 6. *P < 0.05, **P < 0.01, ***P < 0.001.

Next, we evaluated how RBM17 increases T-CA levels. We observed that RBM17-OE can promote the gene expression level of cysteine sulfinate decarboxylase (CSAD) (Fig. 5A). CSAD increases the synthesis of taurocholic acid by promoting taurine synthesis and its binding with bile acids. We hypothesized that RBM17 may enhance CSAD gene expression by altering AS events of CSAD, thus promoting the synthesis and secretion of T-CA. RBM17-OE leads to exon exclusion in CSAD, specifically resulting in the loss of the third exon and the inclusion of the second exon (Fig. 5B, Table S1). We conducted PCR experiments and found that overexpression of RBM17 resulted in the deletion of the third exon and the addition of a second exon in the precursor mRNA of CSAD (Fig. 5C). To verify the splicing specificity of RBM17 for CSAD, we selected splicing factors SRSF1 and HNRNPA1, which have similar functions to RBM17, and constructed cell lines overexpressing SRSF1 and HNRNPA1, respectively. We found that overexpression of SRSF1 or HNRNPA1 did not lead to the exclusion of exon 3 and the inclusion of exon 2 in the CSAD precursor mRNA (Fig. S5A). In addition, overexpression of RBM17 markedly elevated the expression of CSAD protein (Fig. 5D), whereas mutation of its RNA recognition motif (RRM) significantly attenuated this effect (Fig. S5B). Correlation analysis also showed a positive correlation between CSAD expression and taurine metabolism in HCC (Fig. 5E). The use of siRNA targeting CSAD can significantly inhibit RBM17-induced T-CA production (Fig. 5F). This effect has also been observed in in vivo experiments (Fig. 5G). In vitro experiments also observed that overexpression of RBM17^ΔRRM^ could not induce T-CA production (Fig. S5C). FCM detection found that the infiltration of M2 macrophages induced by RBM17 can be reversed by siCSAD (Fig. 5H). Our findings demonstrate that RBM17 enhances T-CA levels by modulating CSAD gene expression through alternative splicing events, leading to increase T-CA metabolism and M2 macrophage infiltration, which are significantly inhibited by targeting CSAD both in vitro and in vivo.

### RBM17 modulates fatty acid metabolism and CD8^+^ T cell infiltration through regulation of exon skipping in HACD3 precursor mRNA

**Fig. 6:**
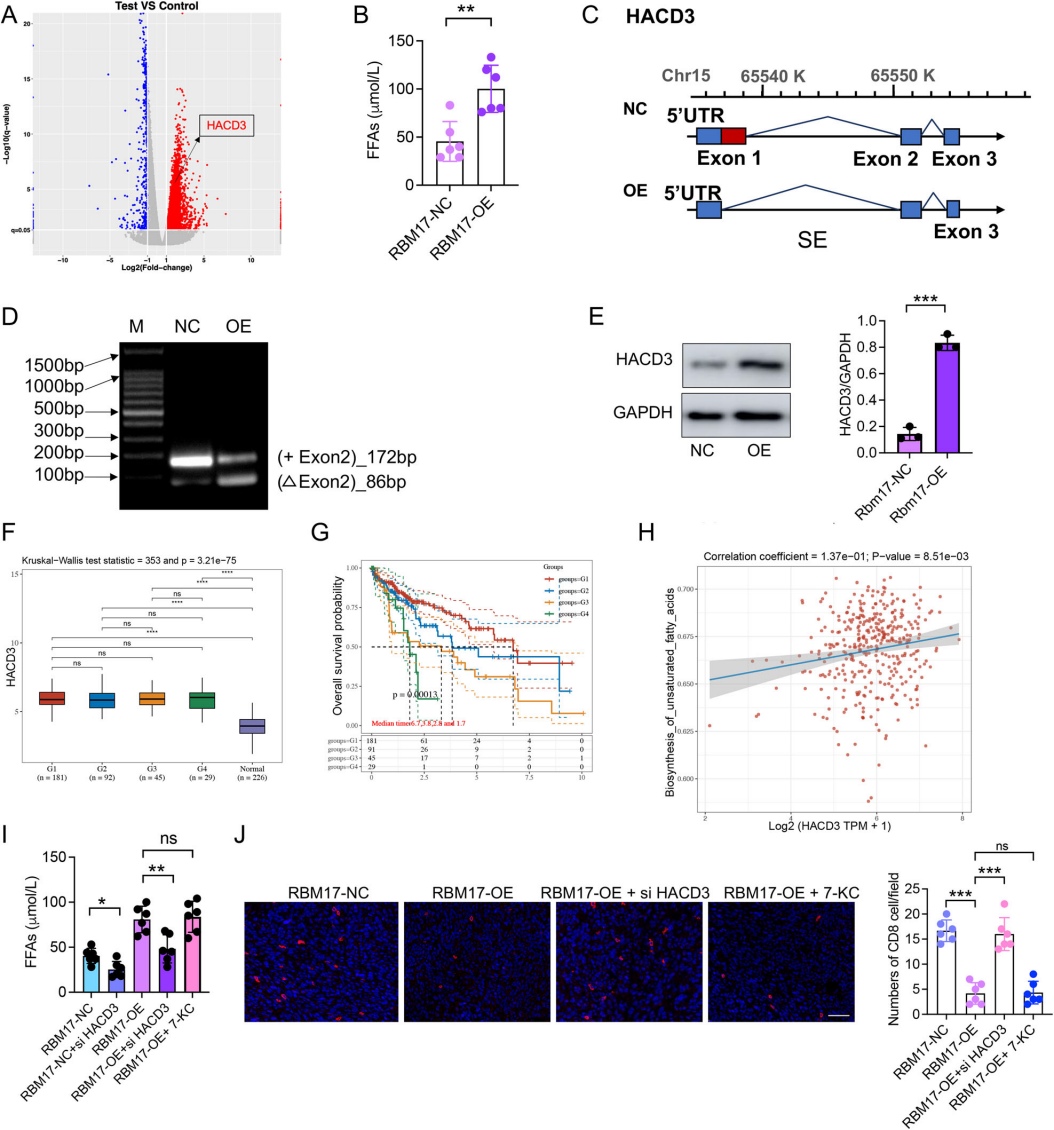
RBM17 increases FFA secretion and reduces the number of CD8^+^ T cells by promoting HACD3 expression. **A** Volcano plot of differentially expressed genes between HepaRG/RBM17-NC and HepaRG/RBM17-OE cells. **B** The effect of RBM17-OE on serum FFA concentration was analyzed in a mouse subcutaneous HCC model. Data was analyzed using unpaired *t-*tests. *n* = 6. **C** The HACD3 gene undergoes exon skipping after overexpression of RBM17. **D** PCR validation of HACD3 AS events. **E** Immunoblot detection of protein expression levels ofHACD3. Data was analyzed using unpaired *t-*tests. *n* = 3. **F**, **G** Comparison of HACD3 expression in cancer versus normal tissues from the TCGA-LIHC dataset, and analysis of the correlation between HACD3 expression and survival rates of HCC patients. **H** Spearman correlation analysis on the relationship between HACD3 expression and FA in HCC. **I** Comparing the effects of siHACD3 and 7-KC on serum FFA concentration in mice through in vivo experiments. Data was analyzed using *t-*tests. ns represents no significant difference. *n* = 6. **J** Immunofluorescence detection of CD8^+^ T cells count in subcutaneous HCC tissue. Bar = 50 µM. Data was analyzed using unpaired *t-*tests. ns represents no significant difference. *n* = 6. *P < 0.05, **P < 0.01, ***P < 0.001.

The above results indicate that fatty acid (FA) metabolism is significant in the AS events influenced by RBM17. We also discovered that the expression of very-long-chain (3 R)-3-hydroxyacyl-CoA dehydratase 3 (HACD3), a pivotal enzyme responsible for converting long-chain FA, was markedly enhanced (Fig. 6A). RBM17-OE accelerated the production of free fatty acid (FFA) (Fig. 6B). Thus, we further explored the relationship between RBM17 and FFA. RBM17-OE led to exon skipping in HACD3, particularly in the first exon (Fig. 6C, Table S1). Subsequently, we conducted PCR experiments and found that RBM17-OE resulted in an 86 bp jump in the first exon of the precursor mRNA of HACD3 (Fig. 6D), but neither SRSF1-OE nor HNRNPA1-OE has this effect (Fig. S5D). In addition, RBM17-OE significantly promoted the expression of HACD3 protein (Fig. 6E), but this promoting effect was significantly inhibited by RBM17^ΔRRM^ (Fig. S5B). We also extracted primary parenchymal cells from mice liver tissue and found that the expression level of CSAD and HACD3 protein were significantly upregulated by RBM17 overexpression (Fig. S6).

Analysis of the TCGA-LIHC dataset revealed that the expression level of HACD3 was significantly upregulated in HCC tissues and was associated with poor prognosis in patients (Fig. 6F, G). Correlation analysis also showed a positive correlation between HACD3 expression and FA synthesis in HCC (Fig. 6H). RBM17-induced FFA was inhibited by siHACD3 but not by 7-KC (Fig. 6I). These results indicated that the T-CA secretion and FFA secretion induced by RBM17 are independent of each other. An increase in FFA in the tumor immune microenvironment is negatively correlated with the activation of CD8^+^ T cells [17]. Correlation analysis showed the expression level of HACD3 was negatively correlated with the infiltration level of CD8^+^ T cells, but not with the infiltration level of M2 macrophages in HCC (Fig. S7). Through the conduct of immunofluorescence and FCM experiments, we discovered that overexpression of Rbm17 significantly inhibits the infiltration of CD8^+^ T cells, and this effect can be reversed by siHACD3 instead of 7-KC (Fig. 6J, Fig. S8A). In addition, we analyzed RNA-seq data and found that overexpression of RBM17 can significantly promote the expression of immunosuppressive cytokines TGFB2 and VEGFA (Fig. S8B). qRT-PCR showed that overexpression of RBM17 promotes transcription of *TGFB2* and *VEGFA* (Fig. S8C). ELISA analysis suggests that overexpression of RBM17 promotes the secretion of TGF-β2 and VEGF, and siHACD3 and siCSAD do not affect this effect (Fig. S8D). These results indicate that RBM17 also promotes the formation of an immunosuppressive microenvironment by activating TGF-β2 and VEGF expression. Therefore, our data demonstrated that RBM17 increases FFA secretion by promoting HACD3 expression, leading to a decrease in the number of CD8^+^ T cells in the TME of HCC. RBM17 also promotes the formation of an immunosuppressive microenvironment by promoting the expression of TGF-β2 and VEGF.

### RUNX1 activates RBM17 expression and regulates downstream CSAD/T-CA and HACD3/FFA signaling

**Fig. 7:**
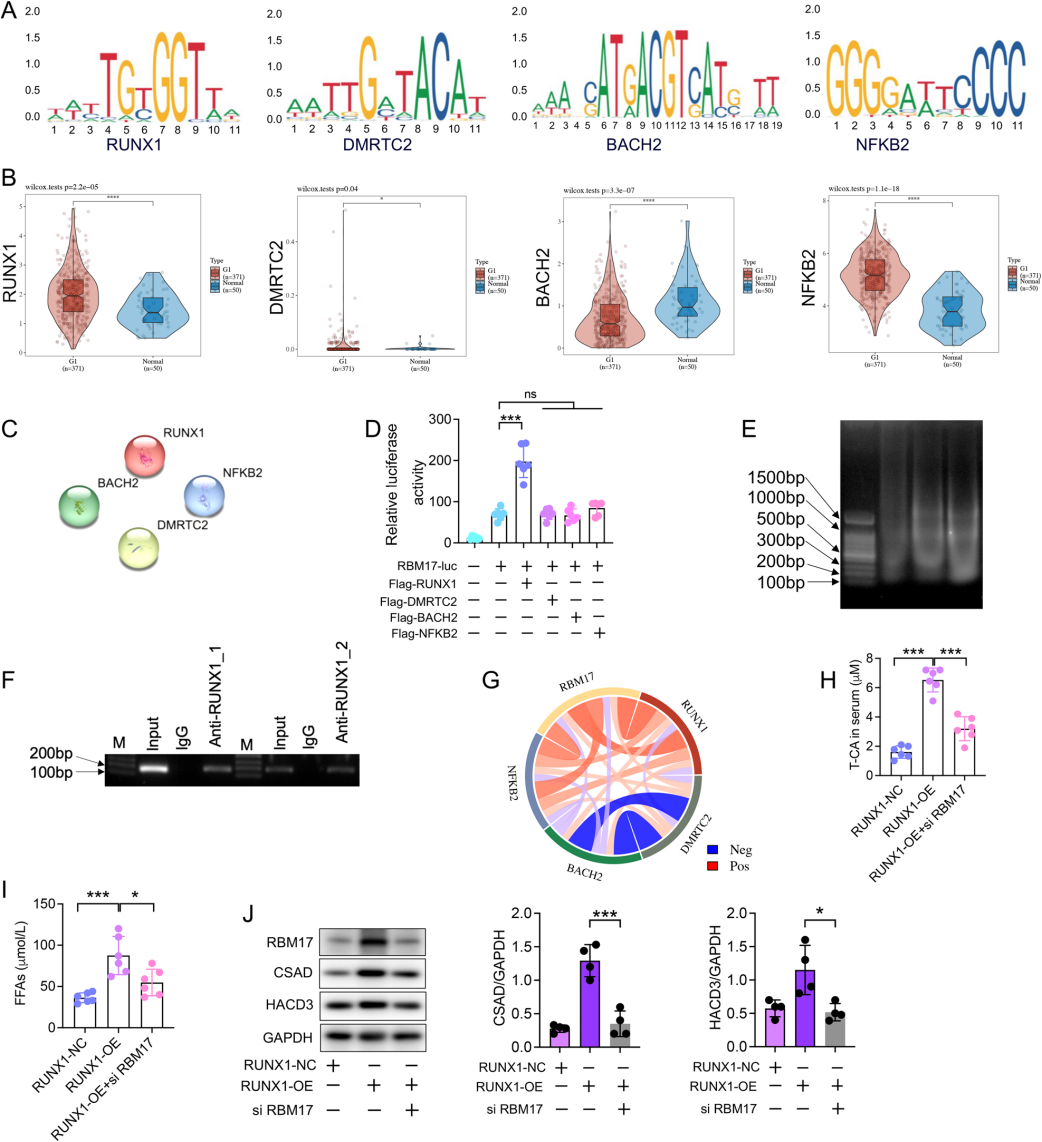
RUNX1 initiates RBM17 expression and controls the regulatory cascades of downstream CSAD/T-CA and HACD3/FFA signaling. **A** Using the JASPAR website (http://jaspar.genereg.net/) and UCSC website (http://genome.ucsc.edu/), RUNX1, DMRTC2, BACH2, and NFKB2 were identified as potential transcription factors for RBM17 and their predicted binding sequences. **B** Expression analysis of RUNX1, DMRTC2, BACH2, and NFKB2 in normal liver tissue and liver cancer tissue. **C** Analyzing the interactions between RUNX1, DMRTC2, BACH2, and NFKB2 using the STRING online website. The interaction score was set at 0.700. **D** The plasmids (pRL-TK, RBM17-luc, Flag-DMRTC2, Flag-RUNX1, Flag-BACH2 and Flag-NFKB2) were transfected into 293 T cells. DLR detects the activity level of RBM17-luc. Data was analyzed using unpaired *t-*tests. ns represents no significant difference. *n* = 6. **E**, **F** We extracted cellular genomic DNA and conducted Chip experiments. **G** Multi gene correlation. In the diagram, red represents positive correlation, blue represents negative correlation, and darker colors indicate stronger correlation between the two. **H** LC‒MS detection of the serum T-CA concentration in mice with subcutaneous HCC. The data were analyzed using *t-*tests. *n* = 6. **I** Comparing the effects of RUNX1 overexpression and siRBM17 on serum FFA concentration in mice through in vivo experiments. Data was analyzed using *t-*tests. *n* = 6. **J** Immunoblot detection of protein expression levels of HACD3, RBM17, CSAD and HACD3. Data was analyzed using *t-*tests. *n* = 3. *P < 0.05, **P < 0.01, ***P < 0.001.

To investigate how RBM17 is overexpressed in HCC cells, we identified RUNX1, DMRTC2, BACH2 and NFKB2 as potential transcription factors for RBM17 using data from the JASPAR website (http://jaspar.genereg.net/) and the UCSC website (http://genome.ucsc.edu/) (Fig. 7A). In addition to BACH2, the expression of RUNX1, DMRTC2 and NFKB2 were significantly upregulated in HCC tissues (Fig. 7B). We employed STRING online analysis to assess the interaction scores among RUNX1, DMRTC2, BACH2 and NFKB2 and found that these four proteins had low interaction scores, suggesting that they may independently regulate RBM17 expression (Fig. 7C). Dual luciferase reporter (DLR) experiments demonstrated that the RBM17 promoter could be activated by RUNX1 but not by the other three transcription factors, indicating that RUNX1 may activate RBM17 expression by binding to its promoter (Fig. 7D). We mutated the predicted RUNX1 binding site in the RBM17 reporter (Mut-RBM17-Luc). Compared with the wild-type RBM17-Luc reporter gene, we observed no significant difference in reporter luminescence in cells transfected with Mut-RBM17-Luc with or without overexpression of RBM17 (Fig. S9). Chromatin immunoprecipitation (Chip) also confirmed the binding of RUNX1 to the RBM17 promoter (Fig. 7E, F). Correlation analysis revealed a significant positive correlation between RUNX1 and RBM17 expression (Fig. 7G). Furthermore, we generated a RUNX1-overexpressing cell line (Hep53.4/RUNX1) and conducted in vivo experiments, which confirmed that the production of T-CA and FFA induced by RUNX1 overexpression could be inhibited by siRBM17 (Fig. 7H, I). Immunoblotting revealed that overexpression of RUNX1 significantly promoted the expression of CSAD and HACD3, while the use of siRBM17 could interfere with this effect (Fig. 7J). The above results suggest that RUNX1 serves as a transcription factor for RBM17, activating RBM17/CSAD/T-CA and RBM17/HACD3/FFA signaling.

### Targeting RBM17 prevents HCC progression

**Fig. 8:**
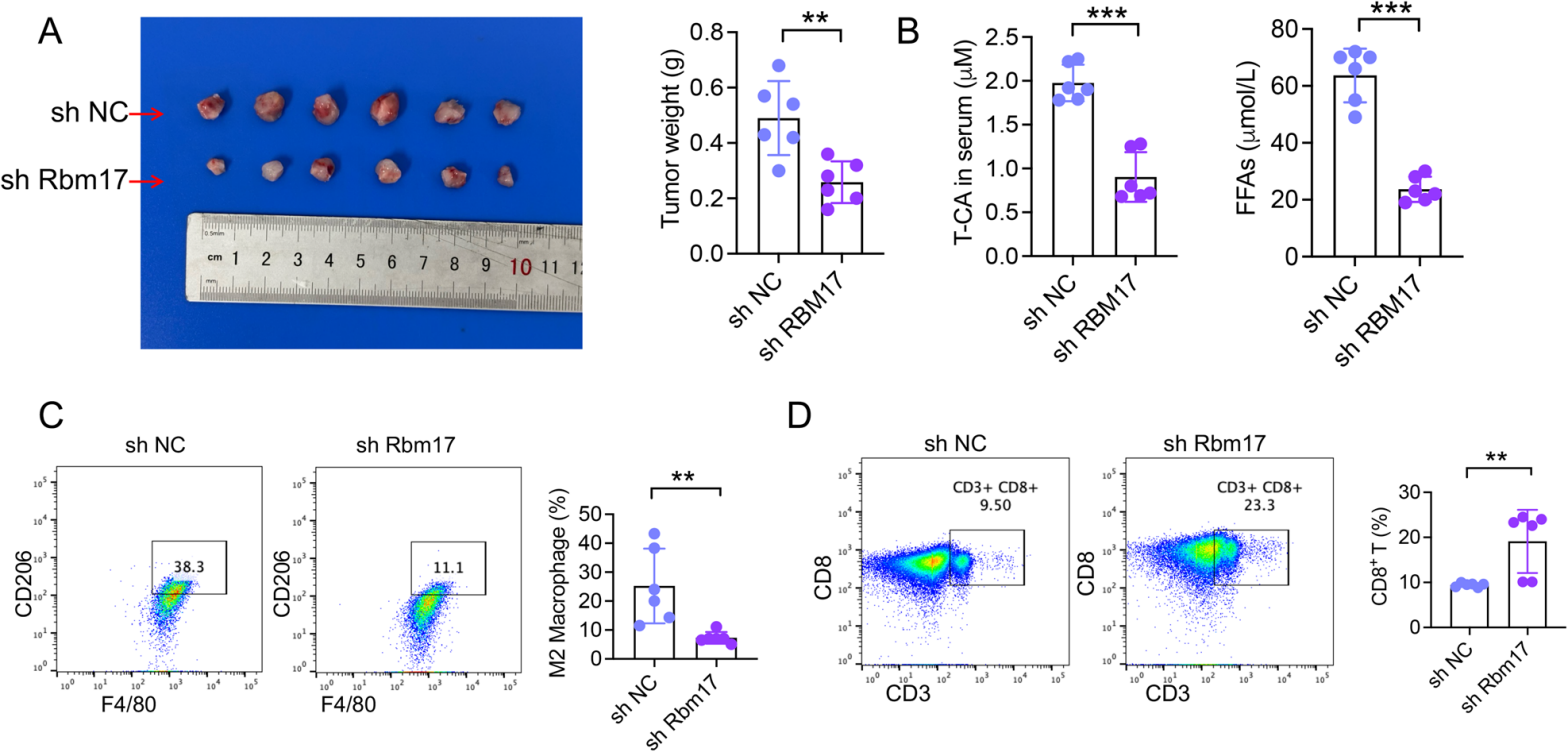
RBM17 promotes HCC growth through RBM17/CSAD/T-CA and RBM17/HACD3/FFA signaling. **A** Tumor tissues were collected. Differences in tumor weight were compared. Data was analyzed using unpaired *t-*tests. ns represents no significant difference. *n* = 6. **B** LC‒MS detection of the serum T-CA and FFA concentration in mice with subcutaneous HCC. The data were analyzed using *t-*tests. *n* = 6. **C**, **D**The infiltration level of M2 macrophages and CD8^+^ T cells in the subcutaneous HCC model was determined using FCM. The data were analyzed using *t-*tests. *n* = 6. *P < 0.05, **P < 0.01, ***P < 0.001.

We next evaluated the effect of RBM17 on HCC growth. We established a subcutaneous HCC model. Knocking down of RBM17 inhibited HCC growth (Fig. 8A). Knocking down RBM17 also restricted the secretion of T-CA and FFA in mice (Fig. 8B). This result confirmed that RBM17 promotes HCC growth through RBM17/T-CA and RBM17/FFA signaling. FCM showed that knockdown of RBM17 inhibited the infiltration of M2 macrophages in tumor tissue, but promoted the infiltration of CD8^+^ T cells (Fig. 8C, D, Fig. S10). These results confirm that RBM17 promotes HCC growth through RBM17/CSAD/T-CA and RBM17/HACD3/FFA signaling.

## Discussion

Recently, increasing emphasis has been placed on individualized treatment for HCC [18]. Targeting AS factors helps to improve the efficacy of tumor treatment [19]. Previous studies have shown that AS factors affect HCC cell proliferation, apoptosis, and migration by regulating gene expression [20, 21]. For example, the AS factor SNRPD2 exhibits aberrant overexpression in HCC, where it drives intron retention in DDX39A pre-mRNA, thereby stabilizing the expression of a functionally distinct short isoform [22]. SRSF1 also exhibits aberrant overexpression and functions as a pro-oncogenic splicing regulator by promoting the generation of the functionally antagonistic KLF6 splice variant 1 (SV1). This splicing switch is mechanistically linked to constitutive activation of the PI3K/AKT signaling cascade, thereby creating a pro-tumorigenic feedback loop [23]. Furthermore, SRSF3 acts as an oncogenic driver during HCC progression by orchestrating a complex splicing network that modulates multiple malignancy hallmarks [24]. Knocking out RBM17 inhibits HCC cell proliferation [25]. Moreover, downregulation of RBM17 enhances the inhibitory effect of cisplatin on pharyngeal squamous carcinoma cells and inhibits cell migration and invasion by reversing EMT [26]. Our study confirmed that the expression of RBM17 is upregulated in HCC tissues and that high expression of RBM17 is associated with poor prognosis in HCC patients. In addition, it has been reported that the AS factor SF3B1 can directly affect the expression of HCC antigens, thereby regulating the antitumor immune effects [27]. Therefore, AS factors play an important role in regulating the biological functions of HCC cells.

AS factors regulate tumor cell metabolism reprogramming [19]. Our study also confirmed that RBM17 promotes the secretion of T-CA and enhances FFA secretion in HCC cells. However, the regulatory effect of RBM17 on T-CA and FFAs is independent. RBM17 performs MEX and SE splicing events on the precursor mRNA of CSAD and HACD3, respectively. We did not find any impact of RBM17 on the splicing events of enzymes related to T-CA metabolism (such as CYP7A1, CYP8B1, CYP27A1, BAAT, BACS, and SULT1A1) and fatty acid metabolism (such as FASN, ACSL1, HACD2, ACC1, ACC2, PNPLA2). This emphasizes the importance of the two independent pathways, RBM17/CSAD/T-CA and RBM17/HACD3/FFA. The AS factor PTBP1 induces the transformation of pyruvate kinase M1 (PKM1) to M2 (PKM2) and promotes glycolysis in breast cancer cells by participating in the cleavage of pyruvate kinase precursor mRNA [8]. Recent studies have shown that the synergistic regulation of the AS factor SF3B3 and RNA binding protein RALY leads to a decrease in MTA1-S levels, enhances the expression of cholesterol synthesis genes, and promotes HCC cell proliferation [10]. Our research also indicates that RUNX1 can bind to the promoter of RBM17 to activate RBM17 expression and maintain its role in lipid metabolism reprogramming. Due to the influence of different metabolites on different biological functions, the effects of RBM17 on other metabolic pathways in HCC cells also require in-depth research.

Lipid metabolism is closely related to tumor immunity. Abnormal BAs in the HCC microenvironment induce tumor immune escape by recruiting NKT cells and M2 macrophages [28]. High concentrations of BAs in serum are independent risk factors for HCC patients [29]. BAs can regulate the immune response by acting on G protein-coupled bile acid receptor (TGR5) and nuclear hormone receptor farnese-like X receptor (FXR) [30, 31]. T-CA promotes IL-4-induced macroscopic M2 polarization by acting on FXR [16]. Obeticholic acid (a derivative of BAs) promotes the secretion of CXCL16 and IFN-γ by endothelial cells, increases the number of NKT cells in the microenvironment, enhances antitumor immunity, and inhibits HCC growth [32]. We discovered that RBM17 induces M2 macrophage infiltration by promoting the secretion of T-CA. Specifically, RBM17 enhances FFA secretion by upregulating HACD3 expression, resulting in a reduction in the population of CD8^+^ T cells, emphasizing that the importance of RBM17 contribute to the promotion of HCC immunosuppressive microenvironment. However, our research requires more human HCC cohorts to support it, especially in terms of confounding factors such as viral hepatitis status or treatment history. In addition, there is a lack of RBM17 knockout mouse experiments to directly confirm the causal role of RBM17 in HCC progression. Recently, multiple drugs targeting SF3B1 have been identified, including FR901464 and its derivatives (eg, spliceostatin A) [33]. We believe that the development of natural drugs or inhibitors targeting RBM17 has therapeutic prospects.

## Conclusions

Our study underscores the critical role of the RBM17 factor in HCC tumor metabolism and growth. By promoting the secretion of T-CA and FFA, RBM17 enhances the infiltration of M2 macrophage but inhibits the infiltration of CD8^+^ T cells, ultimately promoting immunosuppression. This discovery underscores the significance of RBM17 in tumor immunology and growth, suggesting its potential as a target for future therapeutic strategies.

## Materials and methods

### Reagents

The following reagents were obtained from the respective manufacturers: 7-ketocholesterol (7-KC) (MedChemExpress, CAS 566-28-9, China), Taurocholic acid (MedChemExpress, CAS 81-24-3, China), Dulbecco’s Modified Eagle Medium (Gibco, Grand Island, NY), penicillin-streptomycin (Solarbio, Beijing, China), Lipofectamine 3000 (Invitrogen, Carlsbad, CA), Phosphate-buffered saline (PBS) (Solarbio, CAS P1020), puromycin (Solarbio), Nonesterified Free fatty acids assay kit (Jiancheng Bioengineering Institute, Nanjing, China), Type IV (Collagenase IV) (Sigma-Aldrich, CAS C5138), Mouse VEGF ELISA Kit (MultiSciences Biotech, Hangzhou, EK283), Mouse TGF-β2 ELISA Kit (MultiSciences Biotech, Hangzhou, EK9162), Cell Staining Buffer (Biolegend, CAS 420201), Deoxyribonuclease I (DNase I) (Sigma-Aldrich, CAS DN25), Tumor Dissociation Kit (Miltenyi, CAS 130-096-730), and CD45 (TIL) MicroBeads (Miltenyi, CAS 130-110-618). Diethylnitrosamine (DEN, CAS 55-18-5) was obtained from Shanghai Acmec Biochemical Co., Ltd.

### Clinical specimens

The Fujian Cancer Hospital provided HCC tissue samples, which were used for this study after approval from the Ethics Committee of Fujian Cancer Hospital (Approval No. K2024-481-01). Tumor stage and grade were determined based on the eighth edition of the American Joint Committee on Cancer criteria.

### Gene expression analysis

We acquired data from the TCGA database (https://portal.gdc.cancer.gov) by downloading STAR counts data and corresponding clinical information for liver cancer. The TPM format data was then extracted and normalized using log2 (TPM + 1). After filtering, we retained samples with both RNA-seq data and clinical information, yielding a total of 369 samples for further analysis. Statistical analysis was conducted using R software version 4.0.3, with statistical significance determined by a *P*-value of <0.05.

### Prognostic analysis

We obtained STAR-counts data and accompanying clinical information pertinent to LIHC tumors from the TCGA database. Subsequently, we extracted the data in TPM format and normalized it using the log2 (TPM + 1) transformation method. After filtering the samples to ensure they contained both RNA-seq data and clinical information, we narrowed down our selection to 369 samples for in-depth analysis. Our initial step involved performingmultivariate Cox proportional hazards regression analyses. Utilizing the ‘forestplot’ package in R, we created a forest plot to visually depict the *P*-value, hazard ratio (HR), and 95% confidence interval (CI) for each variable.

### Spearman correlation analysis between gene and pathway scores

We obtained RNA-seq data for LIHC and corresponding clinical information from the Cancer Genome Atlas (TCGA) database. We then collected the genes contained in the corresponding pathways and analyzed them using the R software GSVA package, selecting the parameter method = ‘ssgsea’. Finally, we analyzed the correlation between genes and pathway scores using spearman correlation analysis. The analysis method and R software package were both executed using v4.0.3 version R software. *P* < 0.05 was considered statistically significant.

### Cell lines

The human hepatocellular carcinoma cell line Hep3B and the murine HCC cell line Hep53.4 were cultured in Dulbecco’s modified eagle medium (DMEM) supplemented with 10% fetal bovine serum (FBS) and 100 U/mL penicillin/streptomycin. HepaRG cells were obtained from Shanghai Guandao Biological Engineering Co., Ltd., and maintained in RPMI 1640. The cells were maintained at 37 °C in a 5% CO_2_ incubator to ensure standardized growth conditions. Routine mycoplasma contamination tests were conducted on the cell lines, confirming their freedom from mycoplasma contamination.

### Establishment of plasmids and stably transfected cells

Plasmids encoding Myc-tagged RBM17 (Myc-RBM17), Myc-tagged RBM17 lacking the RNA recognition motif (Myc-RBM17^ΔRRM^), human RBM17 overexpression (OE), SRSF1-OE, HNRNPA1-OE, mouse Rbm17-OE, human RBM17-targeting shRNA, and mouse Runx1-OE were obtained from Tsingke Biotechnology Co., Ltd. For cellular transfection experiments, target cells were transfected with the respective shRNA or overexpression plasmids using lipid-based transfection reagents. Following a 48 h incubation period to allow plasmid expression, transfected cells were subjected to selective pressure *via* a 2 day culture supplemented with 3 μg/mL puromycin to establish stable transfectant populations.

### siRNA transfection

siRNA targeting human CSAD, mouse RBM17, CSAD and HACD3 were obtained from GenePharma Corporation (Shanghai, China). According to the instructions, we introduced an appropriate amount of siRNA into cells through transfection reagents. After 36 h, we used Western blotting to detect the protein expression levels of RBM17, CSAD and HACD3.

### Co-cultivation system

To establish a coculture system for tumor cells and THP-1 cells, we inoculated THP-1 cells (2 × 10^6^) into a 6-well plate. Subsequently, PMA (100 ng/mL) was used to induce THP-1 cells to differentiate into macrophages. Next, we inoculated HepaRG/RBM17-NC and HepaRG/ RBM17-OE cells into a 6-well plate, and after 48 h, the HepaRG/ RBM17-OE cells were treated with 7-KC (10 μM) for 24 h. The obtained cell supernatant was filtered and co cultured with PMA-induced THP-1 cells for 24 h. FCM detection of CD206 expression levels.

### Animal model assay

Male C57BL/6 N mice, aged 6–8 weeks and weighing ~17−21 grams, were procured from Shanghai SLAC Laboratory Animal Co., Ltd. Rag1-KO (Rag1-EGFP) mice were purchased from Shanghai Model Organisms Center, Inc. All animals were maintained in a specific pathogen-free (SPF) environment, with the temperature and humidity controlled at 21–23 °C and 40–55%, respectively.

To establish a subcutaneous tumor model, 6-week-old male C57BL/6 N or Rag1-KO mice were randomly distributed into distinct experimental groups. The cells (1 × 10^7^) were then injected subcutaneously into the left flank of each mouse. After a period of 24 days to allow tumor growth, a 0.3% solution of pentobarbital sodium was administered intraperitoneally to induce anesthesia in the mice. Then, the mice were humanely euthanized via cervical dislocation. The resulting tumor tissues were then excised for subsequent in-depth analysis.

The DEN-induced HCC model used was based on our previous research [34]. In brief, 4 week-old male C57BL/6 N mice were intraperitoneally injected with DEN (50 mg/kg, dissolved in PBS) once a week for 32 weeks. The animal experiments were carried out according to the Institutional Animal Care and Use Committee guidelines and were approved by the Experimental Animal Ethics Committee of Fujian Medical University (IACUC FJMU 2024-0343).

### Extraction of primary cells from mouse liver tissue

We used collagenase digestion and density gradient centrifugation to obtain primary parenchymal cells of liver tissue. Briefly, after anesthetizing the mice, we inserted a plastic catheter into the portal vein and perfused the liver with PBS. Subsequently, the liver tissue was cut into small pieces and dispersed in 5 mL of digestion solution (containing 0.1% type IV collagenase, 1% HEPES, 0.5% DNaseI, and 1% FBS), and digested at 37°C for 30 min at 250 r/min. After digestion, filter the cell suspension through a 70 μM filter. To enrich parenchymal cells, the cell suspension was centrifuged at speeds of 200, 500, 800, and 1500 r/min at 4°C. Discard the supernatant and rinse the cells three times with D-Hank solution.

### PCR validation of CSAD and HACD3 AS events

Amplification was carried out as follows: 3 min at 95 °C; 35 cycles of 30 s at 95 °C, 30 s at 55 °C, and 20 s at 72 °C; and 10 min at 72 °C. PCR products were loaded onto a 2% agarose gel. The primers were obtained from Tsingke Biotechnology Co., Ltd. and were as follows: F-CSAD-Exon2: CCTTAGCCATTAGAGAGAGGTCC; F-CSAD-Exon3: GAGCTACCTGTGCACCCTG; R-CSAD-Exon4: CTTCTGGGAGACACTGGTTCC; F-HACD3: CCTGGGCAGTGTGGCC; R-HACD3: GTCTCCTTTGGCACCATGTC.

### qRT‒PCR

Total RNA was extracted from cells using TRIzol reagent (Thermo Fisher Scientific, Waltham, MA). Complementary DNA (cDNA) was synthesized from the total RNA using the RevertAid First Strand cDNA Synthesis Kit (Thermo Fisher Scientific). Quantitative real-time PCR (qPCR) was performed on a QuantStudio 3 Real-Time PCR System (Thermo Fisher Scientific) with FastStart Universal SYBR Green Master Mix (Roche, Mannheim, Germany). Relative mRNA expression levels were calculated using the 2^-ΔΔCt^ method and normalized to the housekeeping gene GAPDH. Primers were synthesized by Tsingke Biotechnology Co., Ltd. (Beijing, China), and their sequences are as follows: 5ʹ-GCAAAGTTGTGAAAACAAGAGC-3ʹ and 5ʹ-ATCCCAGGTTCCTGTCTTTTTATG-3ʹ for *TGFB2*; 5ʹ- GAGGGCAGAATCATCACGAAG-3ʹ and 5ʹ-TGTGCTGTAGGAAGCTCATCTCTC-3ʹ for *VEGFA*; 5ʹ-AGCATGCAGATCAGACCGTG-3ʹ and 5ʹ-GTGAGTTCAGTTTTCTCCCTGC-3ʹ for *FXR*; 5ʹ-GCAAAAAGGGCAACACCACT-3ʹ and 5ʹ-AGGCACATCACTTTCTGAGGG-3ʹ for *CD206*; and 5ʹ- ACACCCACTCCTCCACCTTT -3ʹ and 5ʹ-TCTTCCTCTTGTGCTCTTGCT-3ʹ for *GAPDH*.

### Enzyme-linked immunosorbent assay (ELISA)

Mouse serum was collected. The serum was separated by centrifugation at 4000 rpm for 10 min. We used commercially available ELISA kits and manufacturer’s instructions to measure the levels of mouse TGF-β2 and VEGF.

### Multiparametric immunofluorescence (mIF) staining

We performed multiparametric immunofluorescence staining using a four-color multiple fluorescence immunohistochemical staining kit (RS0035, Immunoway) based on the tyramide signal amplification (TSA) technique, strictly adhering to the manufacturer’s protocol. The resulting images were then precisely captured using a fluorescence microscope.

### Dual luciferase reporter (DLR)

The pGL3-RBM17-Luc reporter construct, its mutant counterpart (pGL3-Mut-RBM17-Luc), and the control pGL3-basic vector were acquired from Tsingke Biotechnology Co., Ltd. For luciferase reporter assays, cells were co-transfected with either the wild-type pGL3-RBM17 promoter-luciferase reporter or the mutant pGL3-Mut-RBM17 construct, along with the pGL3-basic vector using Lipofectamine 3000 transfection reagent. To normalize transfection efficiency and control for experimental variability, the pRL-TK plasmid (Promega) encoding Renilla luciferase was included as an internal reference standard. 48 h post-transfection, cellular lysates were prepared and subjected to Dual-Luciferase Reporter (DLR) analysis following the manufacturer’s protocol (Promega).

### Immunohistochemistry (IHC)

For IHC assays, all sections were boiled in sodium citrate buffer for 10 min, followed by a 25 min incubation in 3% hydrogen peroxide. Subsequently, after being blocked in 1% goat nonimmune serum for 60 min, the sections were incubated with primary antibodies specific for RBM17 (Proteintech, CAS 13918-1-AP, 1:100) at 37 °C for 60 min. After incubation with horseradish peroxidase (HRP)-conjugated secondary antibodies from Affinity Biosciences, the sections were developed using 3,3′-diaminobenzidine.

### Chromatin Immunoprecipitation (Chip)

Formaldehyde was used to fix the DNA-protein complexes in live cells, followed by sonication to obtain small chromatin fragments. Then, antibody (anti-RUNX1, Cell Signaling Tecnology, CAS 12556) specific to the target RUNX1 protein was added to bind to the target protein-DNA complexes, which were subsequently precipitated using magnetic beads. After washing the precipitates to remove nonspecific binding, the crosslinks are reversed and the enriched DNA fragments were purified for PCR to obtain information on protein-DNA interactions. The primers were obtained from Tsingke Biotechnology Co., Ltd. and were as follows: F_1: GAATTGCTGCAGATAATATT; R_1: ATATCAAACACTGTCCCCTA; F_2: GTTTGGTTACGCTTTCTGT; R_2: CCAGAACAAAACTACAAAGC. The amplification reaction process of PCR is: pre denaturation (95 °C, 5 min); denaturation (95 °C, 30 s), annealing (50 °C, 30 s), extension (72 °C, 10 s), 40 cycles.

### Flow cytometry (FCM)

The tumor tissues were digested with a tumor dissociation kit to isolate lymphocytes. Red blood cells were removed by adding 2 mL of lysis buffer and incubating for 15 min. The lymphocyte suspension was layered on top of 5 mL of Ficoll Plus separation buffer (#P4350, Solarbio, Beijing) and centrifuged at 900 × g for 30 min to separate the layers. The cell concentration was adjusted to 2 × 10^5^/mL using staining buffer. Fluorescently labeled primary antibodies were added to the cell suspension and incubated in the dark for 30 min at 4 °C. The expression of target proteins on lymphocytes was detected using a FACS CelestaTM Flow Cytometer (BD Biosciences, NJ, USA). The following antibodies were used: 7-AAD (BD Biosciences, San Jose, CA), BV605-Fixable Viability Stain 575 V (BD Pharmingen), BV650-F480 (BM8, BioLegend, San Diego, CA), APC-CD206 (MR6F3, eBioscience, San Diego, CA), FITC-CD45 (QA17A26, BioLegend), PerCP/Cyanine5.5-CD8 (53–6.7, BioLegend), APC/Cy7-CD3 (17A2, BioLegend), BV421-CD4 (GK1.5, BioLegend) and TruStain FcX™ (anti-mouse CD16/32) antibody (Biolegend).

### Serum FFAs detection

The serum FFAs levels were determined using an assay kit (Jiancheng Bioengineering Institute, Nanjing, China) according to the manufacturer’s instructions.

### Immunoblotting

Cells were plated in 6-cm dishes and lysed in RIPA buffer containing protease inhibitors and PMSF for 15 min at 4 °C. Protein concentrations were measured using a BCA assay. The protein complexes were resolved on SDS‒PAGE gels and transferred to PVDF membranes. The membranes were blocked with 5% skim milk for 1 h at room temperature. Immunoblotting was performed using specific primary antibodies overnight at 4 °C, followed by a 1 h incubation with HRP-conjugated secondary antibodies at room temperature. Bands were detected using a hypersensitive ECL kit. The proportions of primary antibodies used were as follows: HACD3 (Thermo Fisher, H00051495-B01P, 1:1000), CSAD (Thermo Fisher, PA5-120240, 1:1000), RBM17 (Proteintech, CAS 13918-1-AP, 1:1000), GAPDH (Cell Signaling Tecnology, CAS 2118, 1:1000).

### 4D label-free quantitative proteomic analysis

4D-DIA quantitative proteomics was performed and analyzed by Shanghai OE Biotech Co., Ltd. The main steps included: protein extraction, digestion and cleanup, LC‒MS/MS analysis, database search and quantification.

### scRNA-seq

The scRNA-seq and bioinformatics analysis analyses were performed by OE Biotech Co., Ltd. (Shanghai, China). The main methods are as follows:

### Data quality control and gene quantification

To ensure data quality and gene quantification, we used 10x Genomics Cell Ranger software (v7.0.1) to analyze the high-throughput sequencing data in fastq format and compared them to the mouse mm10 reference genome. This software identifies barcode and UMI markers to quantify single-cell transcriptome data, providing crucial quality control statistics such as cell counts, gene medians, and sequencing saturation.

#### Gene quantitative quality control and data preprocessing

After Cell Ranger’s initial quality check, we used Seurat (v4.0.0) to further refine the data. We filtered cells based on key metrics such as nUMI, nGene, and mitochondrial content, retaining only those with gene counts >200, UMI counts >1000, log10GenesPerUMI > 0.7, and mitochondrial/RBC gene proportions <5%. We also used DoubletFinder to remove doublets. Finally, we normalized the data using Seurat’s NormalizeData function.

#### Dimension reduction and clustering analysis

We screened for the top 2000 highly variable genes using Seurat’s FindVariableGenes. We then performed dimensionality reduction, leveraging the expression profiles of these genes. The results were visualized in 2D using UMAP, revealing clusters and patterns in the data.

#### Differential genes and enrichment analysis

We employ the FindMarkers function (test. use = presto) from the Seurat package to identify differentially expressed genes. These genes were selected based on a significance threshold of a *P*-value < 0.05 and a fold change >1.5. Additionally, we conducted GO and KEGG enrichment analyses on these differentially expressed genes using hypergeometric distribution tests.

#### Correlation between Rbm17 gene expression and the expression of other genes

We quantified the correlation between the RBM17 gene and other genes at the expression level by calculating the Spearman correlation coefficient (correlation coefficient level classification: 0.9 < /*r*/<1 is highly correlated; 0.7 < /*r* < 0.9 is strongly correlated; 0.4 < /*r* < 0.7 is moderately correlated; 0.2 < /*r* < 0.4 is weakly correlated; 0 < /*r* < 0.2 is extremely weakly correlated or uncorrelated).

#### The correlation between RBM17 expression and immune cell infiltration

We used ggplot2 to plot scatter plots based on the specified gene expression levels (*x*-axis) and cell proportions of different subtypes (y-axis), and color different points based on cell type.

### RNA-seq

RNA-seq and data analysis, including RNA extraction quality inspection, sequencing library construction, machine sequencing, data preprocessing, gene differential expression analysis, and GO and KEGG enrichment analysis of differentially expressed genes were performed by Shanghai Biotechnology Corporation.

### Statistical analysis

Statistical analyses were performed using SPSS version 25.0 software, and the results are presented as the means ± SD from a minimum of three independent experiments. Significant differences were assessed using either Student’s unpaired *t-*tests or one-way ANOVA.

## Supplementary information

Supplementary information

Full and uncropped western blots

## Data Availability

Most of the data and supplementary data have been provided in the article. The scRNA-seq data generated in this study are publicly available in Sequence Read Archive (SRA) at PRJNA880758 (https://www.ncbi.nlm.nih.gov/bioproject/?term=PRJNA880758). The data from unlabeled proteomics and untargeted metabolomics sequencing have been deposited in the OMIX, China National Center for Bioinformation/Beijing Institute of Genomics, Chinese Academy of Sciences (https://ngdc.cncb.ac.cn/omix: accession no. OMIX006841, OMIX010402). Other data can be provided upon reasonable request.
