# Supplementary material for: RBM17 promotes hepatocellular carcinoma progression by regulating lipid metabolism and immune microenvironment: implications for therapeutic targeting
Source: Cell Death Discov. 2025 Jul 23;11:338. doi: 10.1038/s41420-025-02642-2 (PMC12287257; doi:10.1038/s41420-025-02642-2)
Supplement: Supplementary file 1 — Supplementary information [file 41420_2025_2642_MOESM1_ESM.docx]

**Supplementary information for**

RBM17 Promotes Hepatocellular Carcinoma Progression by Regulating Lipid Metabolism and Immune Microenvironment: Implications for Therapeutic Targeting

Supplementary Figures…………………………………………………………………………2

Supplementary Tables……………………..……………………………………………………13


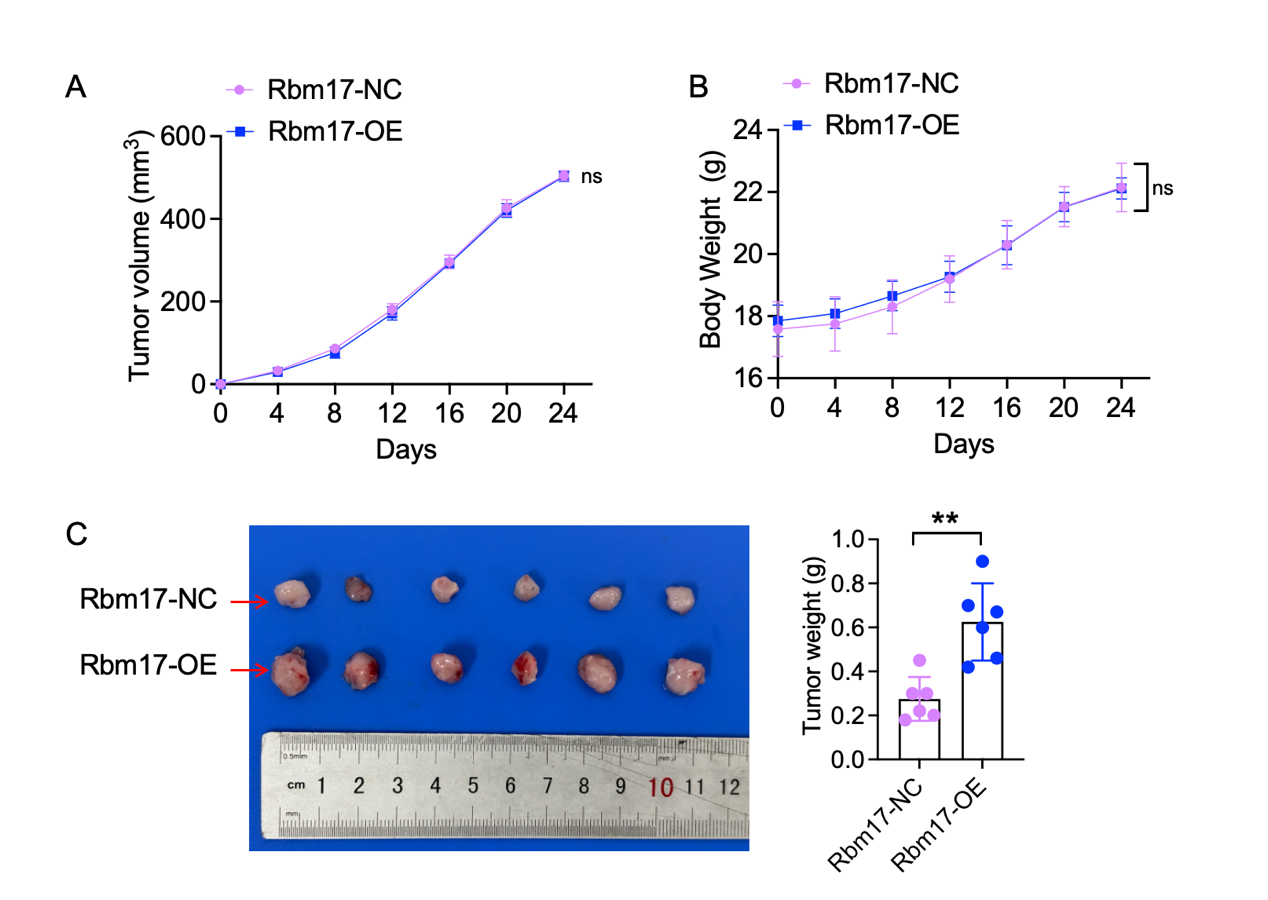


**Figure S1.** The effect of RBM17 overexpression on tumor growth. (A) and (B) Measurement of mouse weight and tumor volume ([W^2^×L]/0.5). We record the weight and tumor volume of mice every 4 days. The data was analyzed using t-test. “ns” represents no significant difference. (C) Tumor tissues were collected. Differences in tumor weight were compared. Data was analyzed using t tests. n=6. ***P* < 0.01.


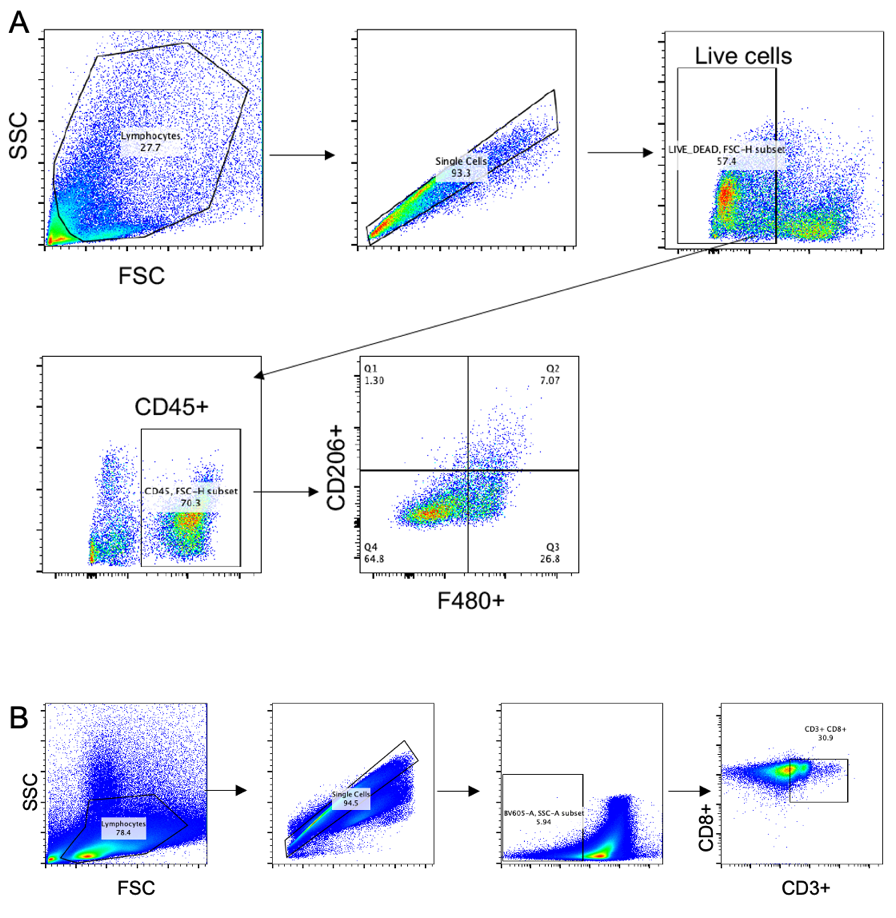


**Figure S2.** Gating strategy for flow cytometry analysis. (A) and (B) Gating strategy for M2 macrophages and CD8^+^ T cells.


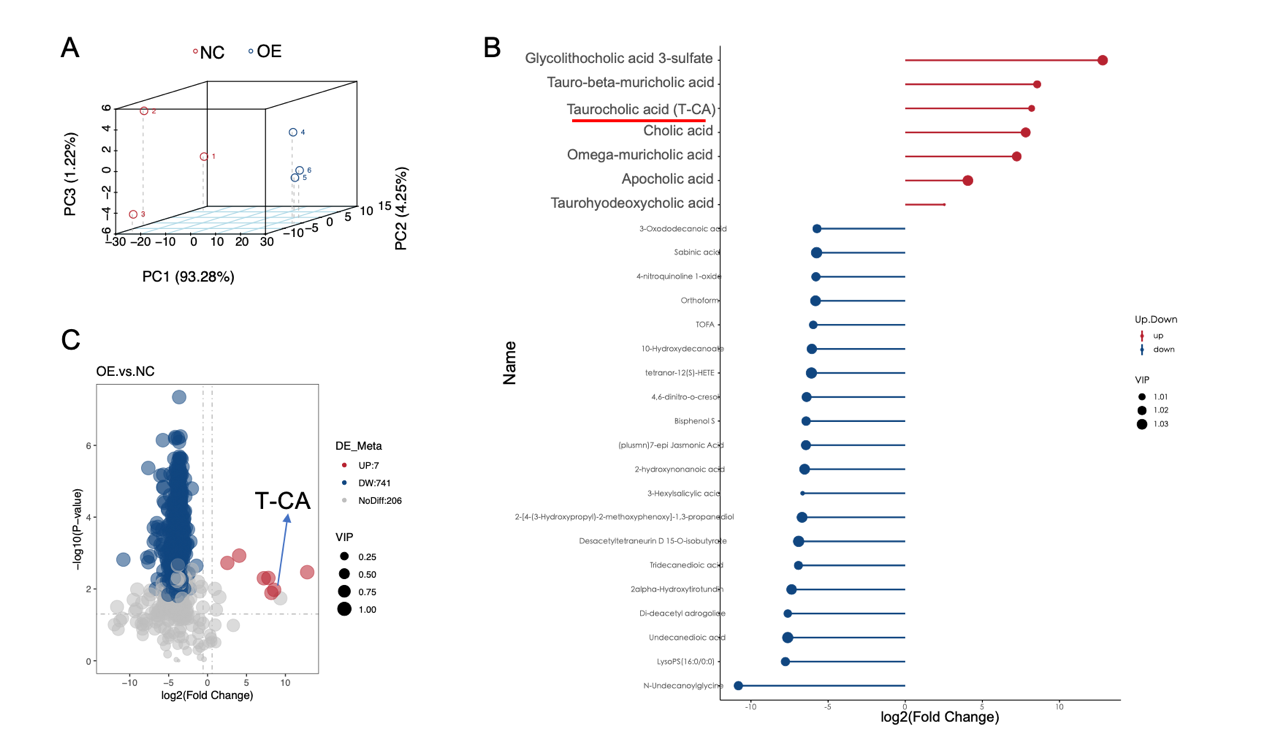


**Figure S3.** Overexpression of RBM17 promotes T-CA metabolism. (A) Total sample PCA analysis. (B) Differential metabolite matchstick diagram. (C) Volcanic diagram of differential metabolites.


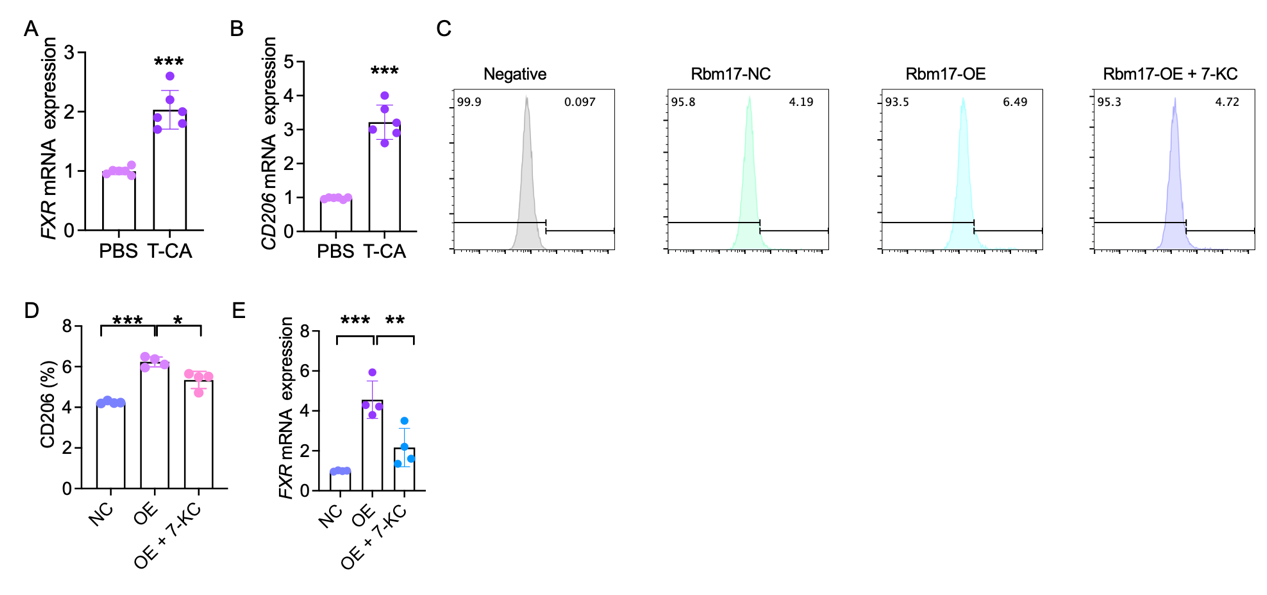


**Figure S4.** The effect of T-CA on M2 polarization and FXR activation of macrophages. (A) and (B) After treating THP1 cells with T-CA (100 μM) for 24 hours, qRT-PCR was used to detect the mRNA levels of *FXR* and *CD206*. n=6. Data was analyzed using t tests. (C) and (D) FCM was used to detect the level of *CD206* in THP1 cells. n=4. Data was analyzed using one-way ANOVA analysis. (E) qRT-PCR was used to detect the mRNA levels of *FXR*. n=4. Data was analyzed using one-way ANOVA analysis. **P* < 0.05, ***P* < 0.01, ****P* < 0.001.


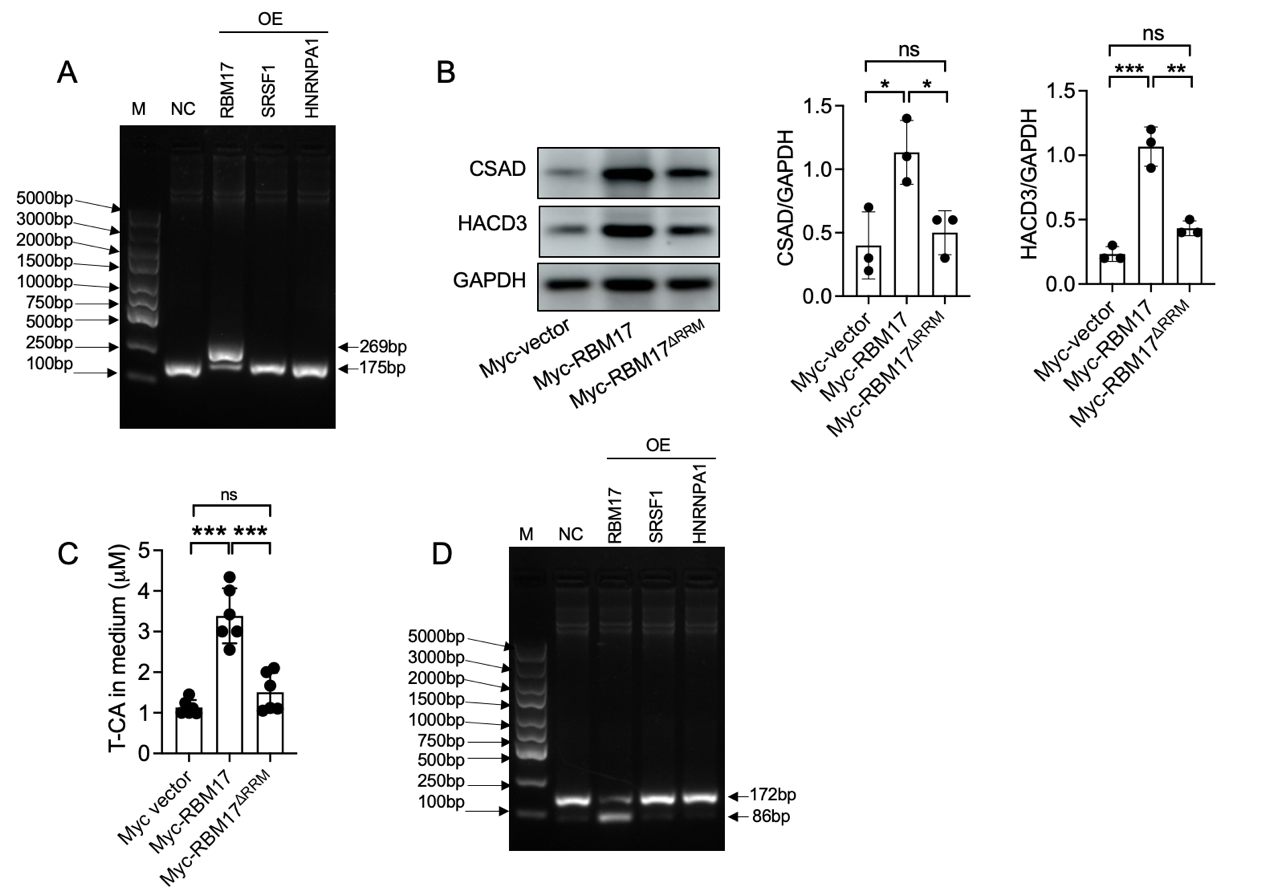


**Figure S5.** Splicing specificity analysis of precursor mRNA of CSAD and HACD3 by RBM17. (A) PCR validation of CSAD AS events. Exon2-Exon4 represents primers designed from the second exon to the fourth exon, while Exon3-Exon4 represents primers designed from the third exon to the fourth exon. (B) Immunoblot analysis of CSAD and HACD3 protein expression levels. Data was analyzed using one-way ANOVA. n=3. (C) LC‒MS detection of the T-CA concentration in cell culture supernatant. The data were analyzed using one-way ANOVA. n=6. (D) PCR validation of HACD3 AS events. **P* < 0.05, ***P* < 0.01, ****P* < 0.001.


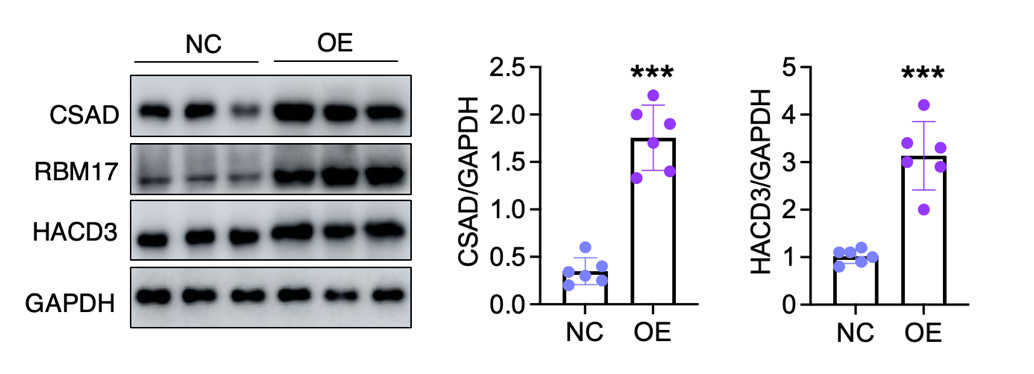


**Figure S6.** The effect of RBM17 overexpression on the expression levels of CSAD and HACD3 proteins. We extracted primary parenchymal cells from mouse liver tissue. We transiently transfected the RBM17 overexpression plasmid into primary parenchymal cells. Immunoblot detection of expression levels of RBM17, CSAD, and HACD3 proteins. Data was analyzed using t tests. n=6. ****P* < 0.001.


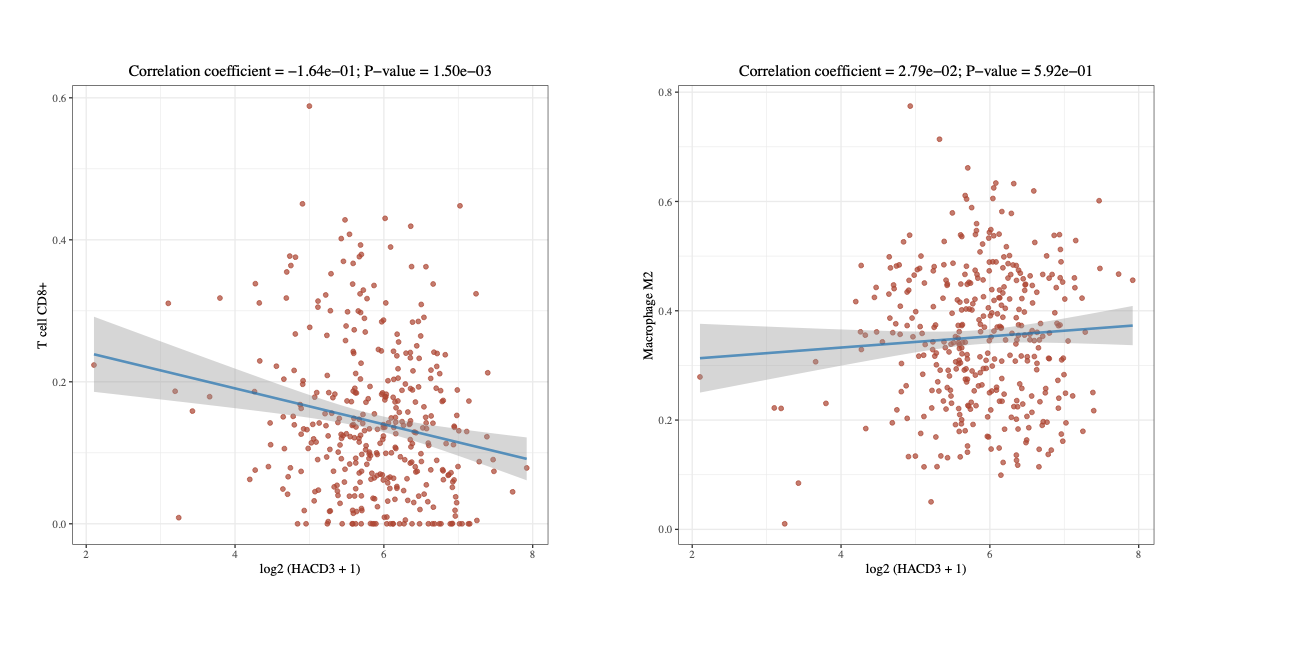


**Figure S7.** Correlation analysis between HACD3 expression and infiltration of CD8^+^ T cells and M2 macrophages. We obtained RNA-seq data and the accompanying clinical details for patients with HCC from the TCGA database. We then subjected these data to analysis using the ggstatsplot package in R software. Spearman correlation analysis was used to evaluate the correlation between HACD3 expression levels and infiltration levels of CD8^+^ T cells and M2 macrophages.


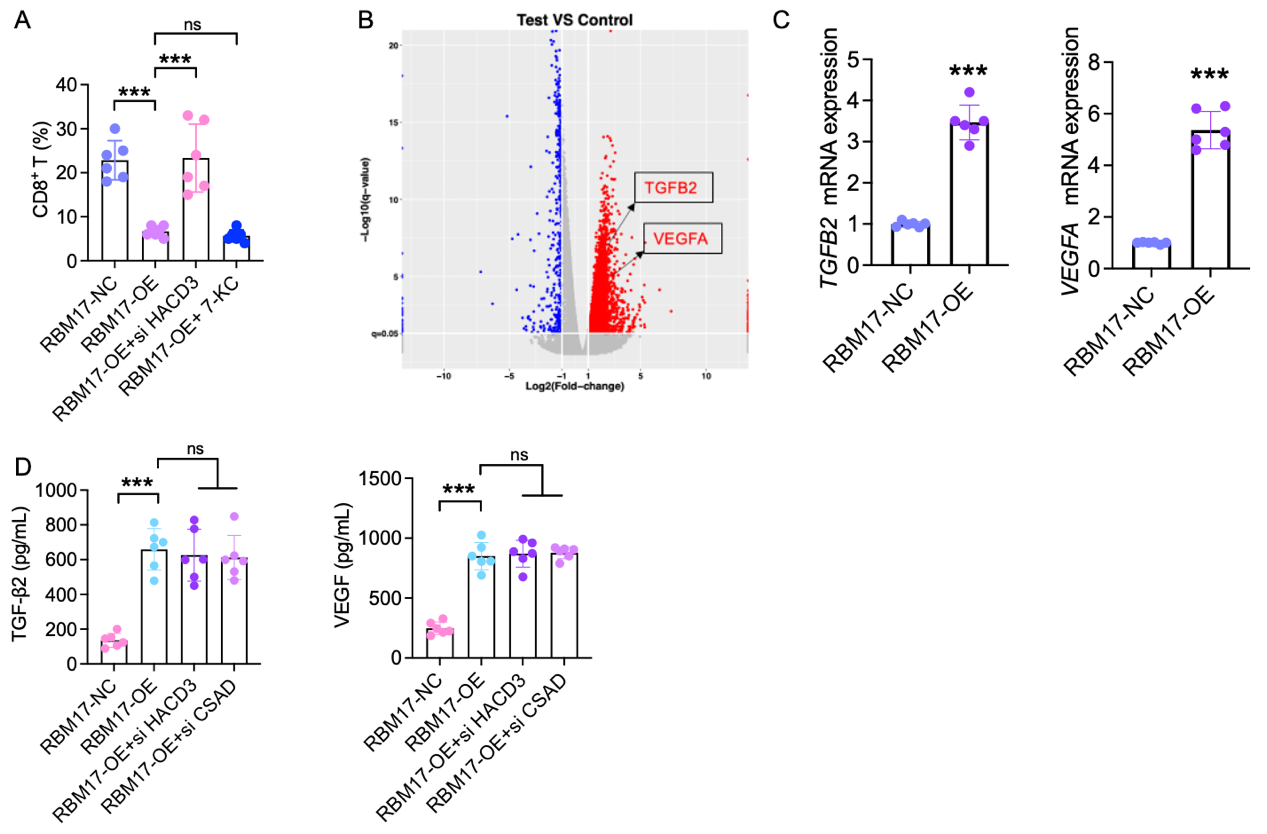


**Figure S8.** RBM17 promotes the formation of an immunosuppressive microenvironment by promoting the expression of TGF-β2 and VEGF. (A) FCM detection of CD8^+^ T cells count in subcutaneous HCC tissue. n=6. Data was analyzed using one-way ANOVA analysis. (B) Volcano plot of differentially expressed genes between HepaRG/RBM17-NC and HepaRG/RBM17-OE cells. (C) qRT-PCR was used to detect the mRNA levels of *TGFB2* and *VEGFA*. n=6. Data was analyzed using t tests. (D) ELISA analysis of serum TGF-β2 and VEGF levels in mice. n=6. Data was analyzed using one-way ANOVA analysis. “ns” represents no significant difference. n=6. ****P* < 0.001.


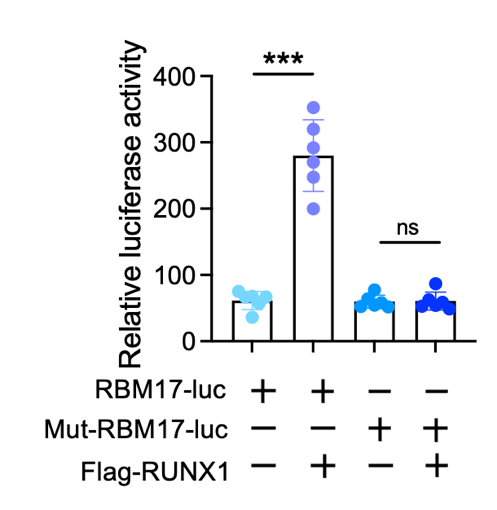


**Figure S9.** Luciferase assay with the PD-L1 promoter was performed in Hep3B cells transfected with or without Flag-RUNX1. The data were analyzed using t tests. n=6. “ns” represents no significant difference. ****P* < 0.001.


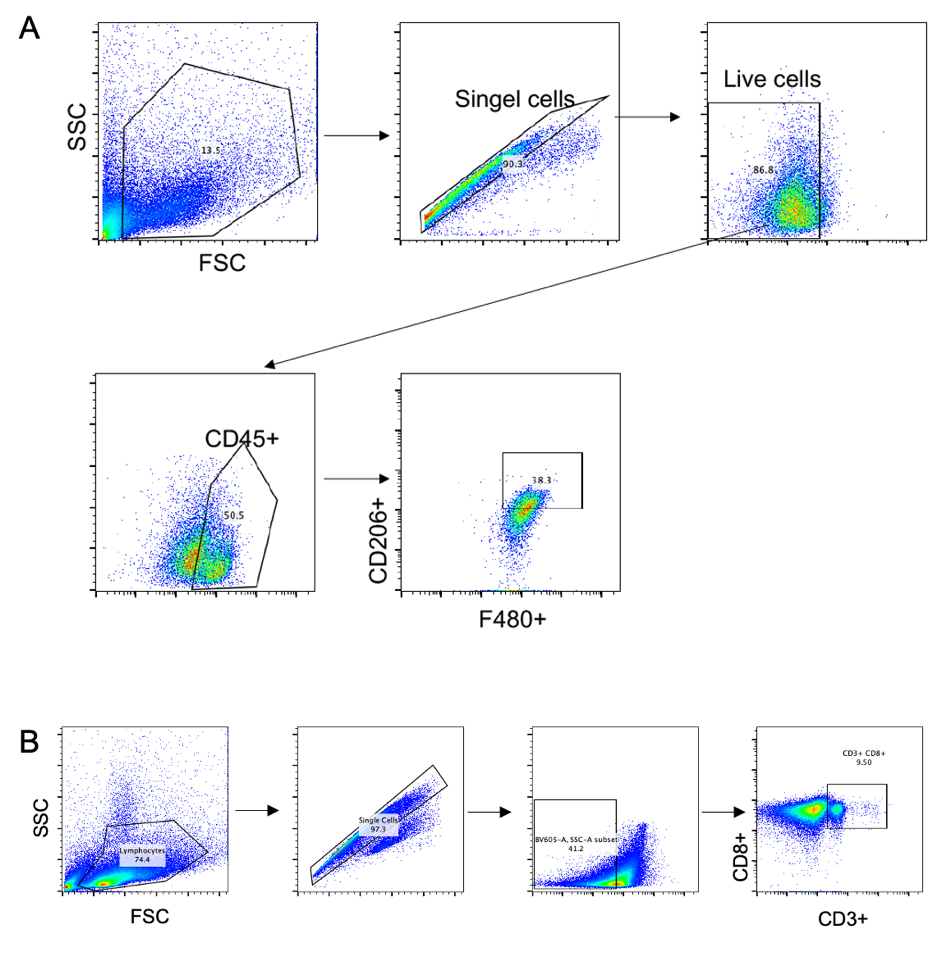


**Figure S10.** Gating strategy for flow cytometry analysis. (A) and (B) Gating strategy for M2 macrophages and CD8^+^ T cells.

**Table S1**

| **event_name** | 15:ENST00000565299-ENST00000562901:65530718-65535721 | 12:ENST00000453446-ENST00000437073:53180601-53173476 |
| --- | --- | --- |
| **Control_posterior_mean** | 0.54 | 0.05 |
| **Control_ci_low** | 0.44 | 0 |
| **Control_ci_high** | 0.63 | 0.17 |
| **Test_posterior_mean** | 0.66 | 0.49 |
| **Test_ci_low** | 0.59 | 0.12 |
| **Test_ci_high** | 0.72 | 0.88 |
| **diff** | 0.12 | 0.44 |
| **bayes_factor** | 1.06 | 2.65 |
| **isoforms** | '15:ENST00000565299-ENST00000562901:65530718-65535721A.1_15:ENST00000565299-ENST00000562901:65530718-65535721A.2','15:ENST00000565299-ENST00000562901:65530718-65535721B.1_15:ENST00000565299-ENST00000562901:65530718-65535721B.2_15:ENST00000565299-ENST00000562901:65530718-65535721B.3' | '12:ENST00000453446-ENST00000437073:53180601-53173476A.1_12:ENST00000453446-ENST00000437073:53180601-53173476A.2_12:ENST00000453446-ENST00000437073:53180601-53173476A.3','12:ENST00000453446-ENST00000437073:53180601-53173476B.1_12:ENST00000453446-ENST00000437073:53180601-53173476B.2_12:ENST00000453446-ENST00000437073:53180601-53173476B.3' |
| **Control_counts** | (0,0):646,(0,1):126,(1,1):342 | (0,0):83,(0,1):8 |
| **Control_assigned_counts** | 0:203,1:265 | 0:0,1:8 |
| **Test_counts** | (0,0):951,(0,1):143,(1,0):1,(1,1):517 | (0,0):122,(0,1):1,(1,0):1 |
| **Test_assigned_counts** | 0:318,1:343 | 0:1,1:1 |
| **chrom** | 15 | 12 |
| **strand** | + | - |
| **mRNA_starts** | 6,553,049,065,530,410 | 5,317,334,553,173,340 |
| **mRNA_ends** | 6,553,583,465,535,830 | 5,318,064,653,180,640 |
| **event_name** | 15:65530490-65530718:65535721-65535834:@65530418-65530718:65534349-65534434:65535721-65535834:+:HACD3 | 12:53173345-53173476:53179773-53180012:53180601-53180646:@53173345-53173476:53173728-53173767:53180601-53180646:-:CSAD |
| **AS_type** | SE | MEX |
| **gene_symbol** | HACD3 | CSAD |
| **gene_id** | ENSG00000074696 | ENSG00000139631 |
