# Supplementary figures and images for: RBM17 promotes hepatocellular carcinoma progression by regulating lipid metabolism and immune microenvironment: implications for therapeutic targeting
Source: Cell Death Discov. 2025 Jul 23;11:338. doi: 10.1038/s41420-025-02642-2 (PMC12287257; doi:10.1038/s41420-025-02642-2)

FIG 2F

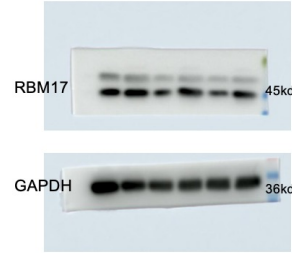

FIG 5D

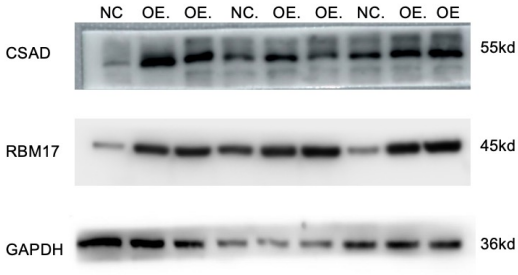

FIG 7J

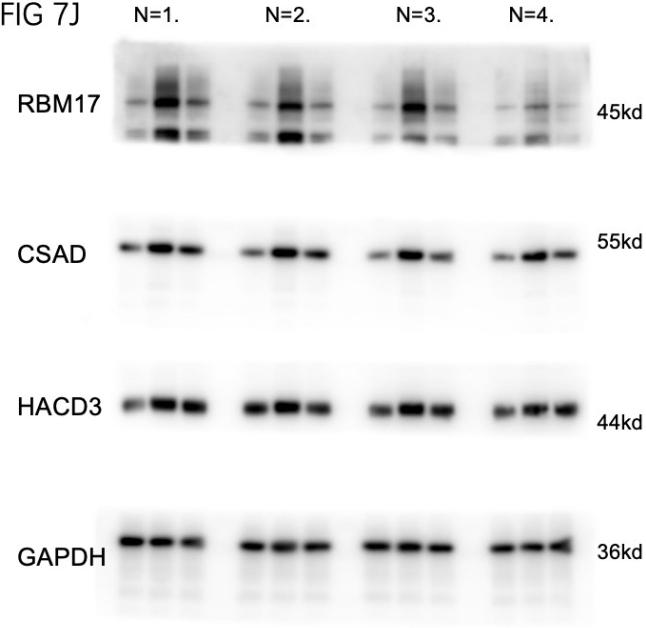

FIG 6E

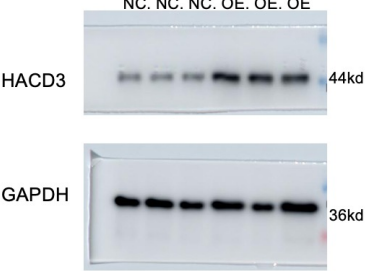

FIG S5B

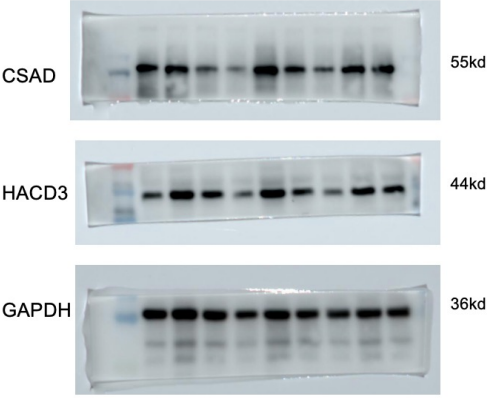

FIG S6

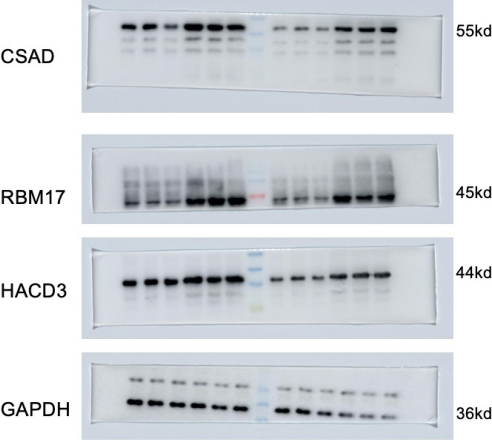

Supplement: Supplementary file 2 — Full and uncropped western blots [file 41420_2025_2642_MOESM2_ESM.pdf]
